# Fluid–structure interaction in a fully coupled three-dimensional mitral–atrium–pulmonary model

**DOI:** 10.1007/s10237-021-01444-6

**Published:** 2021-03-26

**Authors:** Liuyang Feng, Hao Gao, Nan Qi, Mark Danton, Nicholas A. Hill, Xiaoyu Luo

**Affiliations:** 1 School of Mathematics and Statistics, University of Glasgow, Glasgow, G12 8SQ UK; 2 Institute of Marine Science and Technology, Shandong University, Shangdong, 266237 People’s Republic of China; 3 Department of Cardiac Surgery, Royal Hospital for Children, Glasgow, UK

**Keywords:** Pulmonary circulation, Left atrium, Mitral valve, Fluid–structure interaction, Mitral regurgitation, Pulmonary hypertension

## Abstract

This paper aims to investigate detailed mechanical interactions between the pulmonary haemodynamics and left heart function in pathophysiological situations (e.g. atrial fibrillation and acute mitral regurgitation). This is achieved by developing a complex computational framework for a coupled pulmonary circulation, left atrium and mitral valve model. The left atrium and mitral valve are modelled with physiologically realistic three-dimensional geometries, fibre-reinforced hyperelastic materials and fluid–structure interaction, and the pulmonary vessels are modelled as one-dimensional network ended with structured trees, with specified vessel geometries and wall material properties. This new coupled model reveals some interesting results which could be of diagnostic values. For example, the wave propagation through the pulmonary vasculature can lead to different arrival times for the second systolic flow wave (S2 wave) among the pulmonary veins, forming vortex rings inside the left atrium. In the case of acute mitral regurgitation, the left atrium experiences an increased energy dissipation and pressure elevation. The pulmonary veins can experience increased wave intensities, reversal flow during systole and increased early-diastolic flow wave (D wave), which in turn causes an additional flow wave across the mitral valve (L wave), as well as a reversal flow at the left atrial appendage orifice. In the case of atrial fibrillation, we show that the loss of active contraction is associated with a slower flow inside the left atrial appendage and disappearances of the late-diastole atrial reversal wave (AR wave) and the first systolic wave (S1 wave) in pulmonary veins. The haemodynamic changes along the pulmonary vessel trees on different scales from microscopic vessels to the main pulmonary artery can all be captured in this model. The work promises a potential in quantifying disease progression and medical treatments of various pulmonary diseases such as the pulmonary hypertension due to a left heart dysfunction.

## Introduction

The pulmonary circulation system is a complex multiscale network of vessels with diameters ranging from 2 to 3 cm in the main pulmonary artery down to about 10 $\upmu$m in the capillaries (Townsley 2011). It transports the blood received from the right ventricle (RV) to the left atrium (LA) with continuous pulmonary venous outflow (De Marchi et al. 2001) throughout the whole cardiac period. Therefore, it plays an important haemodynamic role in the LA. On the other hand, the LA also directly affects the blood pressure and flow inside pulmonary vessels, especially under pathological conditions. The most common cause of pulmonary hypertension is left heart disease (PHLHD), in particular obstructive left heart disease (Galiè et al. 2016; Guazzi and Borlaug 2012), and the classic lesion that causes this is mitral stenosis and regurgitation (Maeder et al. 2018; Magne et al. 2015). From a clinical standpoint, PHLHD can be, to a degree, reversible if the obstruction is corrected in a timely fashion, e.g. using mitral valve (MV) replacement. However, severe and long-term obstruction triggers structural and functional changes in pulmonary vessels (Shimoda and Laurie 2013) leading to fixed elevation of pulmonary pressure and resistance. As a result, the pulmonary artery pressure remains elevated even when the obstruction is relieved. This highlights the importance of tracking the interaction between the pulmonary circulation and the LA. However, measuring pressure inside small pulmonary vessels (Souza et al. 2005; Takala 2006), pulmonary veins and LA directly is not routinely clinically possible (Galiè et al. 2016).

Common clinical measurements for the pulmonary circulation (Galiè et al. 2016) include vessel anatomy, arterial pressure and flow and pulmonary artery wedge pressure.  These are usually obtained via cardiac tomography, echocardiography, cardiac magnetic resonance and right heart catheterization. However, these medical technologies do not provide important haemodynamic details such as the pulmonary capillary pressure (Gaar Jr et al. 1967; Grimbert 1988), a key determinant of pulmonary oedema (Ware and Matthay 2005) and of the structural changes in the distal arterioles (Shimoda and Laurie 2013), nor do these provide the pulmonary venous pressure, a direct reflection of left heart performance (Dadfarmay et al. 2010), or the travelling waves inside the pulmonary vessel network (Parker and Jones 1990; Parker 2009) which give insights into the link between pulmonary haemodynamics and heart function (Su et al. 2016). Indeed, the shape and magnitude of the travelling waves reflect the RV and LA contractility, vessel stiffness and the resistance of pulmonary vasculature. And it is known that pulmonary venous waves are heavily influenced by the left atrium function (Hellevik et al. 1999; Smiseth et al. 1999; Bouwmeester et al. 2014). However, many previous studies focused on the pulmonary arterial waves (Qureshi and Hill 2015; Hollander et al. 2001; Dwyer et al. 2012; Su et al. 2017; Smolich et al. 2010) and much less attention has been on the venous side (Smiseth et al. 1999; Hellevik et al. 1999; Bouwmeester et al. 2014).

Computational modelling has shown significant value in tracking the normal and diseased behaviour of pulmonary circulation, as it can present detailed information under controlled environments (Tawhai et al. 2011). Both one-dimensional (Kheyfets et al. 2013; Dawson et al. 1999; Bshouty and Younes 1990; Qureshi et al. 2014) or three-dimensional models (Tang et al. 2011; Spilker et al. 2007; Kheyfets et al. 2015) have been developed. Compared with three-dimensional models, one-dimensional models can easily incorporate more complete patient-specific geometry such as vessel length and cross-sectional area and still provide key information such as pressure and flow throughout the entire vasculature and also are less computationally expensive. In the previous research, the pulmonary circulation and left heart are often treated as two independent units with prescribed boundary conditions (Qureshi et al. 2014; Bosi et al. 2018; Masci et al. 2017; Vedula et al. 2015; Koizumi et al. 2015; Masci et al. 2017). However, as aforementioned, the pulmonary circulation and LA have close interactions, especially under pathological conditions. In the case of PHLHD, the disease progression is characterised as the reversible stage in which the pulmonary pressure elevates proportionally to the increased LA pressure, and the irreversible stage (Patel et al. 2014) in which the tissue growth and remodelling appear in both pulmonary vessels and LA walls (Cameli et al. 2017; Messika-Zeitoun et al. 2007) in response to pressure and volume overload. Therefore, it is necessary to incorporate LA models into the modelling of pulmonary circulation to gain more insights into the exact mechanism underlying this process. So far, little work has been done to look into the coupling effects between these two systems using complex computational models.

Lumped parameter model has been traditionally used to study the flow in the pulmonary circulation and the left heart (Shimizu et al. 2018; Liang and Liu 2005; Frolov et al. 2017; Virag and Lulić 2017), and a closed-loop cardiovascular system including both systemic and pulmonary circulation, heart chambers and heart valves can be achieved relatively easy. However, such lumped models do not provide detailed flow patterns inside heart chambers and around valves; this limits their applications if detailed flow quantities and local structural stresses and strains are needed (Feng et al. 2019; Gao et al. 2017a). As previously observed (Fyrenius et al. 2001; Suwa et al. 2014; Kilner et al. 2000; Föll et al. 2013), the inflow of left and right pulmonary veins leads to different contribution to the intra-atrial vortex formation. Furthermore, in the case of mitral regurgitation, the regurgitant jet directions can have different impact on the flow pattern of each pulmonary veins (Pieper et al. 1996; Klein et al. 1991). In order to investigate such behaviour, it is necessary to include physiologically realistic left heart models rather than simple elastance components (Pieper et al. 1996; Klein et al. 1991).

The aim of this study is to investigate the interaction between the pulmonary circulation and the LA using computational modelling tools under control conditions and left heart dysfunctions (acute mitral regurgitation (AMR) and atrial fibrillation (AF)). In particular, we explore the effect of wave propagation in the pulmonary vasculature on the LA pressure and flow field, the early stage haemodynamic changes in both systems caused by incompetent MV leaflets, and the loss of atrial active contraction. The pulmonary circulation is represented by a one-dimensional structured tree model originally developed by Qureshi et al. (2014), which takes into account of large vessel length, cross-sectional area, connectivity, wall material properties, as well as fluid dynamics of both large and microscopic vessels. The LA is modelled using a previously developed coupled hyperelastic LA-MV framework (Feng et al. 2019) using a hybrid immersed boundary—finite element method (Peskin 2002; Griffith and Luo 2017). In this new model, the coupling to the pulmonary circulation is achieved at the outlet of the pulmonary veins where pressure and flow information are exchanged, with a prescribed pressure boundary condition applied at the upstream (RV side) and the downstream (left ventricle (LV) side).

The remainder of paper is organized as follows: details of the pulmonary circulation model and the LA-MV model are presented in Sect. 2, together with the boundary conditions and numerical implementation. Results and discussion are given in Sects. 3 and 4, respectively.

## Methodology

### The pulmonary circulation model

**Fig. 1:**
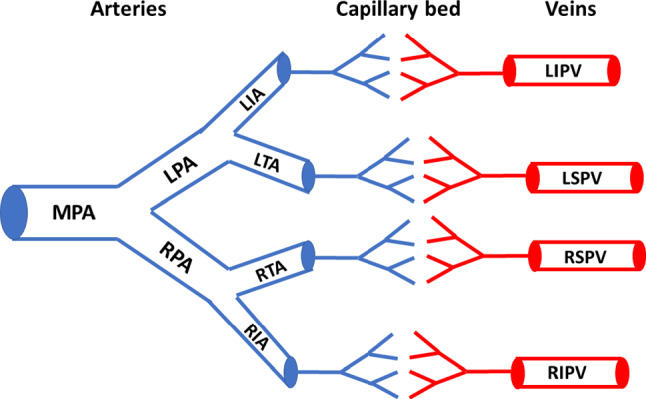
Illustration of the one-dimensional pulmonary circulation model. *MPA* main pulmonary artery, *RPA/LPA* right and left pulmonary arteries, *RIA/LIA* right and left interlobular arteries, *RTA/LTA* right and left trunk arteries, *RIPV/LIPV* right and left inferior pulmonary veins, *RSPV/LSPV* right and left superior pulmonary veins

The one-dimensional pulmonary circulation model developed by Qureshi et al. (2014) includes seven large arteries, four large veins and separate structured tree models for small arterial and venous vessels, as illustrated in Fig. 1. Modifications about the vessel geometry have been made to adapt to the LA-MV model presented in Sect. 2.2 to first ensure matching of the proximal diameters for the four large pulmonary veins between the pulmonary model and the LA-MV model. Next, moving from venous side to arterial side, large vessel length and diameters are modified to maintain the same distal-to-proximal diameter ratio and the length-to-diameter ratio in each vessel, as well as the same diameter-to-diameter ratio at the branching junctions from the original data (Qureshi et al. 2014). Table 1 lists the large vessel geometry information used in the current study. Besides the large vessels, the capillary bed shown in Fig. 1 is modelled as structured trees generated based on several assumptions: firstly each parent vessel connects to two daughter vessels, namely $\alpha$ and $\beta$ branch, whose radius can be predicted via the relation

1 $$\begin{aligned} r_\alpha =\alpha \ r_\text {p},\quad r_\beta =\beta \ r_\text {p}, \end{aligned}$$

where $r_\text {p}$, $r_\alpha$ and $r_\beta$ denote the radius for the parent vessel and two daughter vessels. $\alpha =0.91$ and $\beta =0.58$ are constants derived based on the previous studies (Qureshi et al. 2014; Pollanen 1992). In addition, the length of each vessel is also specified following a length-to-radius ratio relation (Qureshi et al. 2014; Fung 2013). Secondly, the structured tree on the venous side is considered as a ‘mirror-image’ of the corresponding arterial tree in terms of generations. Thirdly, the cut-off value for the radius of the smallest vessels in the structured trees is set to be 30 $\upmu$m in the current study and the vessels with radius less than 30 $\upmu$m such as capillaries are neglected. Different choices of the cut-off value have also been tested, and the comparison is listed in Sect. Parameter sensitivity test in ‘Appendix’. This choice of cut-off value is to ensure that the cardiac parameters, such as the pulmonary artery pressure, pulmonary venous flow, and the LA pressure, fall within the normal physiological range (Tabata et al. 2003; Kane et al. 2016; Blume et al. 2011) for the control case studied here.

**Table 1:** Geometric information (cm) of the large vessels in our model

Name	Prox. diameter	Dist. diameter	Length
MPA	3.1	3.0	4.5
RPA	2.5	1.6	5.8
LPA	1.8	1.8	2.5
RIA	1.5	1.5	1.6
RTA	1.5	1.5	1.7
LIA	1.7	1.5	1.9
LTA	1.4	1.4	1.2
RIPV	1.5	1.5	1.6
RSPV	1.7	1.5	2.5
LIPV	1.6	1.5	1.8
LSPV	1.5	1.4	2.3

The fluid dynamics in the large vessels is governed by the following equations

2 $$\begin{aligned}&\frac{\partial q}{\partial t}+\frac{\partial }{\partial x}\left( \frac{q^2}{A}\right) +\frac{A}{\rho }\frac{\partial p}{\partial x}=-\frac{2\pi \nu R}{\epsilon }\frac{q}{A}, \end{aligned}$$

3 $$\begin{aligned}&\frac{\partial q}{\partial x}+\frac{\partial A}{\partial t}=0, \end{aligned}$$

4 $$\begin{aligned}&p(x,t)-p_0=\frac{4}{3}\frac{Eh}{R_0}\left( \sqrt{\frac{A}{A_0}}-1\right) . \end{aligned}$$

Equation (2) is the momentum equation assuming a flat velocity profile *u*(*r*, *x*, *t*) with a thin boundary layer of constant thickness $\epsilon$

5 $$\begin{aligned} u= {\left\{ \begin{array}{ll} {\bar{u}}, &{} \text {for}\ r\le R-\epsilon \\ {\bar{u}}(R-r)/\epsilon , &{} \text {for}\ R-\epsilon <r\le R \end{array}\right. } \end{aligned}$$

where ${\bar{u}}(x,t)$ is the constant central velocity, *r* is the radial coordinate measured from the centre line of the vessel, and *R*(*x*, *t*) is the vessel radius. *q*(*x*, *t*) and *A*(*x*, *t*) denote the volumetric flow rate and cross-sectional area at location *x* and time *t*. $\nu$ is the kinematic viscosity. Equation (3) is the continuity equation ensuring volume conservation with the assumption of incompressible flow and (4) is a tube law with $p_0$ being the external pressure, *E* is the Young’s modulus, and $\textit{h}$ is the vessel wall thickness. $R_0$ and $A_0$ are the undeformed vessel radius and cross-sectional area. $Eh/R_0= 150.0$ mmHg is used for all the large arteries and veins in the current study due to the lack of experimental data and also motivated by previous studies (Krenz and Dawson 2003; Attinger 1963; Yen et al. 1990) that suggest pulmonary compliance is diameter independent and constant across the system. Variations in the vessel stiffness are also discussed in Sect. Parameter sensitivity test in ‘Appendix’ in which similar results are found.

In the small vessels, under the assumption that the fluid velocity is axisymmetric and $\ll$ the wave speed, and by making the long-wavelength approximation, linearisation of the governing Eqs. (2), (3) and (4) leads to

6 $$\begin{aligned}&\frac{\partial u}{\partial t}+\frac{1}{\rho }\frac{\partial p}{\partial x}=\frac{\nu }{r}\frac{\partial }{\partial r}\left( r\frac{\partial u}{\partial r}\right) , \end{aligned}$$

7 $$\begin{aligned}&C\frac{\partial p}{\partial t}+\frac{\partial q}{\partial x}=0, \end{aligned}$$

8 $$\begin{aligned}&C=\frac{\partial A}{\partial p}\approx \frac{3A_0 R_0}{2Eh}, \end{aligned}$$

where *u*(r,x,t) denotes the flow velocity. The closed-form solutions for Eqs. (6), (7) and (8) can be obtained (Qureshi et al. 2014) under the periodic conditions given observations that the blood flow within the pulmonary capillary bed is pulsatile with the same period as the heartbeat (Reuben 1970; Maloney et al. 1968). The wall stiffness for the small vessels is determined from

9 $$\begin{aligned} \frac{Eh}{R_0}=k_1 \exp {(k_2 R_0)}+k_3, \end{aligned}$$

where $k_1= 187.5$ mmHg, $k_2 = -10.0$ $\hbox {cm}^{-1}$ and $k_3= 37.5$ mmHg (Reuben 1970; Maloney et al. 1968) from the experimental data in Yen et al. (1990) and Yen and Sobin (1988). For more details, readers are referred to Qureshi et al. (2014).

### The LA-MV model

Most of the details of the LA-MV model has been published (Feng et al. 2018, 2019). Here, we describe the model for completeness. The LA model is based on one recently published dataset (Fastl et al. 2018) from a 35-year-old male patient with hyperlipidemia as shown in Fig. 3b. The LA wall is modelled as a combination of passive and active material, i.e.

10 $$\begin{aligned} \mathbb {P}^\text {e}_\text {LA}=\mathbb {P}^\text {e}_\text {LA-passive} + \mathbb {P}^\text {e}_\text {LA-active}, \end{aligned}$$

in which

11 $$\begin{aligned}&\mathbb {P}^\text {e}_\text {LA-passive}\nonumber \\&\quad = a\exp \left( b(I_1 -3)\right) \mathbb {F}-a\exp \left( b(I_1 -3)\right) \mathbb {F}^{-T} \nonumber \\&\qquad + 2a_1(I_4-1)\exp {\left( b_1\left( I_4-1\right) ^2\right) }\mathbb {F}\mathbf{e}\otimes \mathbf{e}\nonumber \\&\qquad +\beta \log (I_3)\mathbb {F}^{-T}, \end{aligned}$$

and

12 $$\begin{aligned} \mathbb {P}^\text {e}_\text {LA-active}=J {T}_{\text {active}}\left( 1+\gamma (\lambda -1)\right) \mathbb {F}\mathbf{e}\otimes \mathbf{e}. \end{aligned}$$

**Fig. 2:**
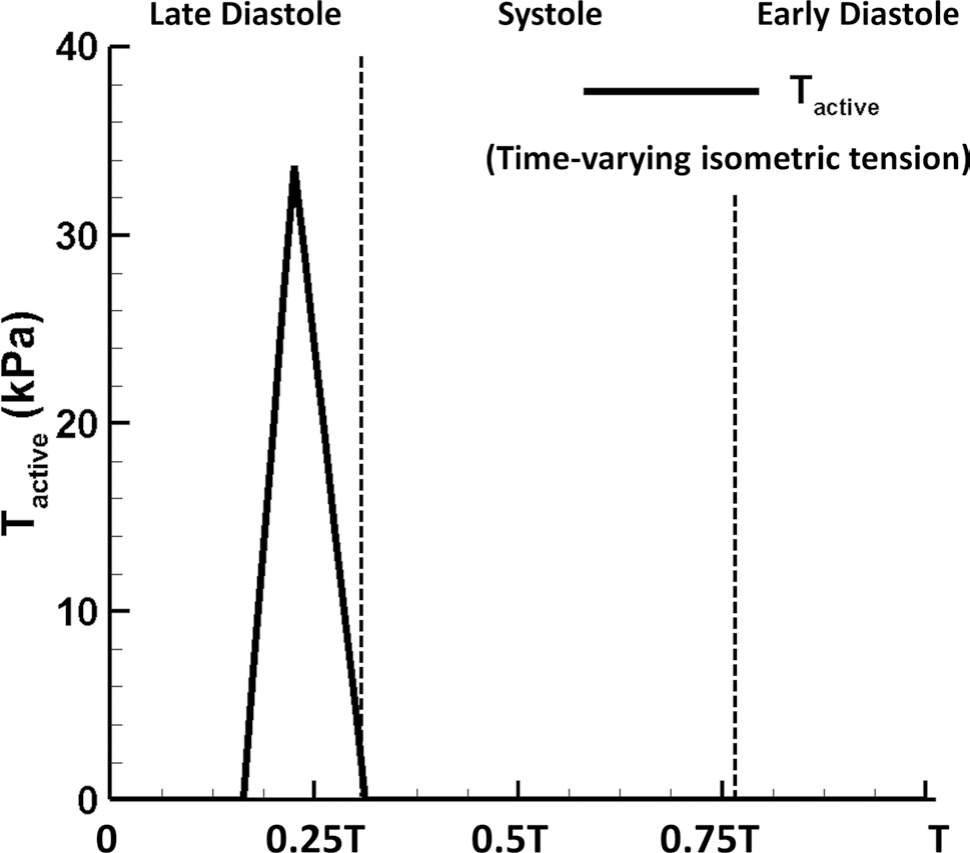
Profile of* T*$_\text {active}$ with the peak value of 33.7 kPa

Here, $\mathbb {P}^\text {e}$ is the elastic stress tensor, $\mathbb {F}$ is the deformation gradient. $I_1=\text {tr}(\mathbb {F}^T\mathbb {F})$ and $I_3=\text {det}(\mathbb {F}^T \mathbb {F})=J^2$. $\mathbf{e}$ denotes the fibre orientation at the reference configuration and $I_{4}=\lambda ^2=\mathbf{e}\cdot (\mathbb {F}^T\mathbb {F})\mathbf{e}$. The term $\beta \log (I_3)\mathbb {F}^{-T}$ is used to ensure incompressibility, with $\beta$ chosen to be 500 kPa. ${T}_{\text {active}}$ is a linear function representing the time-varying isometric tension as shown in Fig. 2 similar to the work by Wang et al. (2010) based on the LV pressure-volume animal data. We further assume the LA contracts homogeneously and simultaneously across the whole LA wall. Note that a more realistic electrophysiology–mechanics coupling can be included in the future for triggering LA contraction (Quarteroni et al. 2017), while this would make the model much more challenging in computation and parameter selection. In terms of the current parameter value selection for the LA material model, the calibration process involves manually rescaling the maximum isometric tension 56.2 kPa reported by Niederer et al. (2006) for ${T}_{\text {active}}$ and rescaling the passive material parameters used by Genet et al. (2016) obtained from the ventricular myocardium data. The goal of the calibration process is to ensure the LA model obtains a typical stroke volume 100 mL per cycle and a normal LA phasic function. For example, the normal flow contribution to the stroke volume for the typical LA phasic function is $27\%$ (passive emptying volume), $18\%$ (active emptying volume) and $55\%$ (conduit volume) according to Wright et al. (2015). The passive material parameters for the LA are listed in Table 2.

**Fig. 3:**
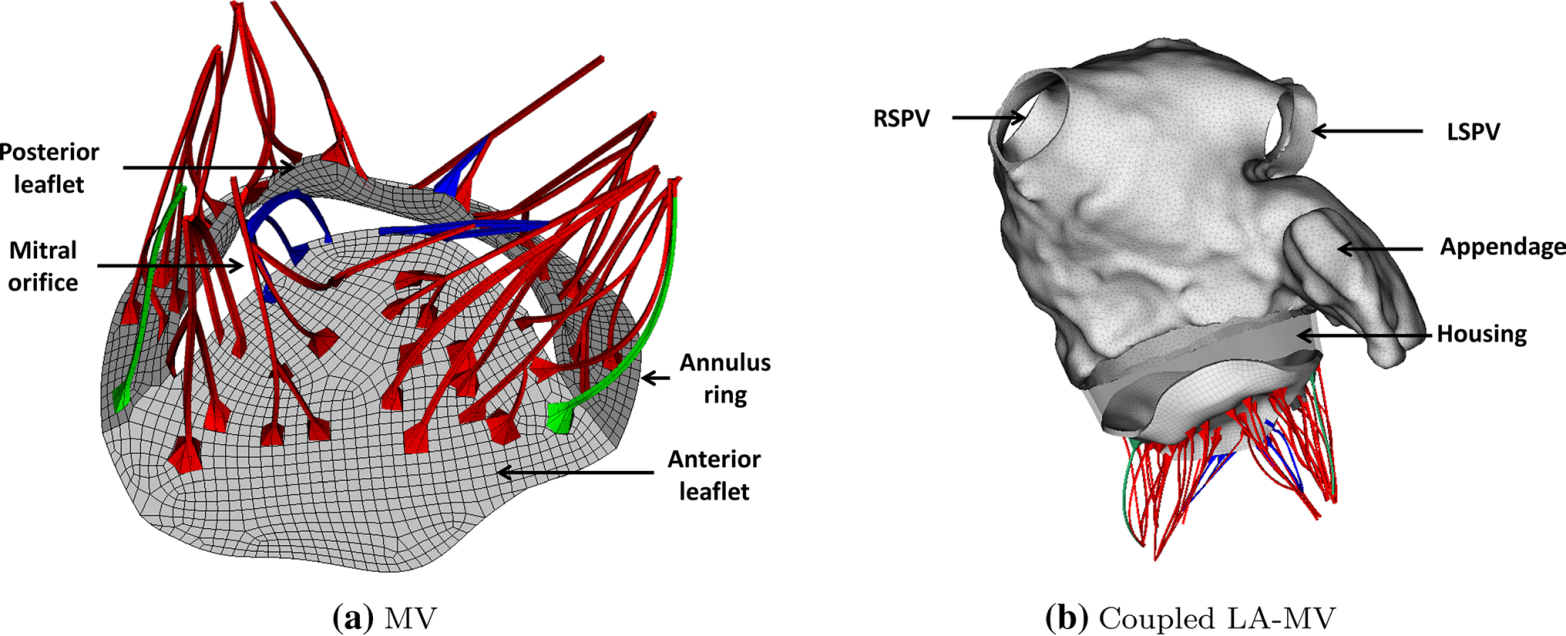
MV geometry (left) with light grey indicating the anterior leaflet and dark grey indicating the posterior leaflet, and the coupled LA-MV geometry (right) with a rigid housing. Red: strut chordae; blue: marginal chordae; green: basal chordae

The MV model in Fig. 3 uses the leaflet and chordae geometry constructed by Wang and Sun (2013), based on multi-slice computed tomography scans of a normal valve from a 61-year-old male patient. The leaflets and chordae are modelled as two different passive materials

13 $$\begin{aligned} \mathbb {P}^\text {e}_\text {leaflet}&= 2C_1C_2\left( \exp (C_2(I_1 -3))\mathbb {F}-\exp \left( C_2(I_1 -3)\right) \mathbb {F}^{-T}\right) \\&\quad+\sum _{i=1}^2 2k_1\kappa (I_{4i}^*-1)\exp {\left( k_2\left( I_{4i}^*-1\right) ^2\right) }\mathbb {F}\\&\quad+\sum _{i=1}^2 2k_1(1-3\kappa )(I_{4i}^*-1)\exp {\left( k_2\left( I_{4i}^*-1\right) ^2\right) }\mathbb {F}\mathbf{e}_i\otimes \mathbf{e}_i\\&\quad+\beta \log (I_3)\mathbb {F}^{-T},\end{aligned}$$

and

14 $$\begin{aligned} \mathbb {P}^\text {e}_\text {chordae}&=2C\mathbb {F}-2C\mathbb {F}^{-T}+2\alpha _1\exp {\left( \alpha _2(I_4 -1)^2\right) }\mathbb {F}\mathbf{e}\otimes \mathbf{e}\\&\quad+\beta \log (I_3)\mathbb {F}^{-T}. \end{aligned}$$

where $I_{4i}^*=\text {max}[(\kappa I_1 +(1-3\kappa )I_{4i}),1]$ in which $\kappa$ is a dispersion parameter used to describe the distribution of fibre orientation (Gasser et al. 2006), and $I_{4i}=\mathbf{e}_i\cdot (\mathbb {F}^T\mathbb {F})\mathbf{e}_i$. Two families of fibres are used for the leaflet material and one family used for the chordae defined along its longitudinal direction (Feng et al. 2018). The MV material parameters’ values are based on the experimental tests from Wang and Sun (2013) and published data from literature (Kunzelman and Cochran 1990) and listed in Tables 3 and 4.

**Table 2:** Material parameters of the LA wall based on the data from Genet et al. (2016)

	*a* (kPa)	*b*	$a_{1}$ (kPa)	$b_1$	$\gamma$
LA wall	8.0	5.75	6.0	4.06	4.9

**Table 3:** Material parameters of the MV leaflets from Wang and Sun (2013)

	$C_1$ (kPa)	$C_2$	$k_{1}$ (kPa)	$k_2$	$\kappa$
Anterior	0.12	13.67	11.01	84.85	0.08
Posterior	0.05	15.00	3.02	144.48	0.053

**Table 4:** Material parameters of MV chordae based on the data from Kunzelman and Cochran (1990)

	*C* (kPa)	$\alpha _1$ (kPa)	$\alpha _2$
Basal chordae	540	1446.2	22.09
Marginal chordae	540	200.48	33.83
Strut chordae	540	1446.2	22.09

For simplicity, the LA and MV models are connected using a rigid housing based on the mitral annular shape although we recognize that the mitral annulus, a fibrous construct, is not a rigid structure and deforms its shape throughout the cardiac cycle. Additionally, rigid tubes representing the four pulmonary veins and left ventricle are constructed to mount the coupled model on the computational domain boundary where pressure and flow boundary conditions are applied. More details of the LA and MV model can be found in Feng et al. (2019).

The full fluid–structure interaction is captured in immersed boundary framework with finite element elasticity (IB/FE) (Griffith and Luo 2017; Gao et al. 2014), in which the structure deformation and elasticity are described in the Lagrangian form, while the fluid velocity and pressure are described in the Eulerian form. Let $\Omega \subset \mathbb {R}^3$ denote the fixed physical domain in which the coupled fluid–structure system sits. $\Omega ^\text {s}_0\subset \Omega$ represents the reference domain for the structure, and $\mathbf{X} \in \Omega ^\text {s}_0$ is the Lagrangian coordinate for any structure material point. $\varvec{\chi }(\mathbf{X},t)\in \Omega$ is an isomorphism mapping of each material point to its current physical position at time *t*. Therefore, $\Omega ^s_t = \varvec{\chi }(\Omega ^s_0,t)$ represents the region occupied by the deformed structure at time *t* and $\Omega ^\text {f}_t = \Omega \backslash \Omega ^\text {s}_t$ is the fluid region.

The governing equations for the coupled LA-MV system are

15 $$\begin{aligned}&\rho \left( \frac{\partial \mathbf{u}(\mathbf{x},t)}{\partial t }+\mathbf{u}(\mathbf{x},t)\cdot \nabla \mathbf{u}(\mathbf{x},t)\right) \\&\quad =-\nabla p(\mathbf{x},t)+\mu \nabla ^2 \mathbf{u}(\mathbf{x},t)+\mathbf{f}(\mathbf{x},t), \end{aligned}$$

16 $$\begin{aligned}&\nabla \cdot \mathbf{u}(\mathbf{x},t)=0, \end{aligned}$$

17 $$\begin{aligned}&\mathbf{f}(\mathbf{x},t)=\int _{\Omega ^\text {s}_0} \mathbf{F}(\mathbf{X},t)\ \delta (\mathbf{x}-\varvec{\chi }(\mathbf{X},t)) \,\text {d}\mathbf{X}, \end{aligned}$$

18 $$\begin{aligned}&\int _{\Omega ^\text {s}_0}\mathbf{F}(\mathbf{X},t)\cdot \mathbf{V}(\mathbf{X}) \,\text {d}\mathbf{X}\\&\quad =-\int _{\Omega ^\text {s}_0}\mathbb {P}^\text {e}(\mathbf{X},t):\nabla _{\mathbf{X}}\mathbf{V}(\mathbf{X}) \,\text {d}\mathbf{X} ,\ \forall \mathbf{V}(\mathbf{X}), \end{aligned}$$

19 $$\begin{aligned}&\frac{\partial \varvec{\chi }}{\partial t }(\mathbf{X},t)=\mathbf{u}(\varvec{\chi }(\mathbf{X},t),t)=\int _{\Omega }\mathbf{u}(\mathbf{x},t)\ \delta (\varvec{\chi }(\mathbf{X},t)-\mathbf{x}) \, \text {d}\mathbf{x}, \end{aligned}$$

Equations (15) and (16) are the momentum equation and the continuity equation, in which $\mathbf{u}(\mathbf{x},t)$, $p(\mathbf{x},t)$ denote the fluid velocity and pressure, and $\rho$, $\mu$ are the constant material density and dynamic viscosity. Both the structure and fluid are assumed to be incompressible in the current study. Equations (17) and (19) represent the classical IB interaction scheme for structural force density and velocity, with the three-dimensional Dirac delta function $\delta (\mathbf{x})$. Equation (18) computes the finite elasticity with the test function ${\bf V}({\bf X})$ and $\mathbb {P}^\text {e}(\mathbf{X},t)$ is the first Piola–Kirchhoff stress tensor.

### Fully coupled LA-MV and pulmonary circulation model

**Fig. 4:**
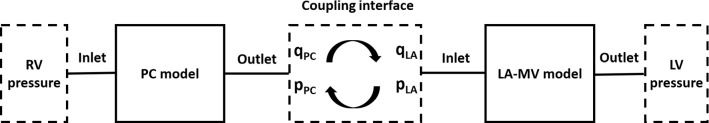
Illustration of the coupled pulmonary circulation, LA and MV model. $q_\text {PC}$, $p_\text {PC}$ are the flow rate and pressure at the outlet of pulmonary veins. $q_\text {LA}$ is the flow rate at the inlet of the LA model and $p_\text {LA}$ is the LA main chamber pressure

The details of the numerical scheme used to solve the coupled system are listed in Sect. Numerical algorithms in ‘Appendix’. The focus here is to explain the coupling of the pulmonary circulation model and the LA-MV model at the connection between the pulmonary veins and the LA chamber. This is similar to the work by Chen et al. (2016), in which an explicit cosimulation scheme was developed that allows each subsystem to use its specialised solver and avoid rewriting everything into one system, as shown in Fig. 4. In the circular cross sections at the ends of the pulmonary veins in the LA-MV model, the velocity is assumed to be that of Poiseuille flow and the pressure is computed numerically.

For the convenience of explanation, we assume here both models use the same time-step size though this can be changed in the numerical implementation. At the *n*th time step, $t^n$, the flow rate and pressure at the coupling interface of the fully coupled model are denoted as $q^n_\text {PC}$, $p^n_\text {PC}$, $q^n_\text {LA}$ and $p^n_\text {LA}$, respectively. Prior to the coupling of the two models, the single LA-MV model is pre-run for an initialization phase of 0.1 s with prescribed pulmonary inflow boundary conditions. Similarly, the single pulmonary model is pre-run for ten cardiac cycles, using a prescribed typical LA pressure boundary condition that has the peak pressure of 6 mmHg for the ‘a’ wave and 9 mmHg for the ‘v’ wave, to converge to a steady stage to ensure $q_\text {PC}$ = $q_\text {LA}$ and $p_\text {PC}$ = $p_\text {LA}$ when the coupling takes place. After the two models are coupled, the time-stepping scheme is as follows. First, the flow rate in the LA-MV model for the next time step $t^{n+1}$ is predicted via

20 $$\begin{aligned} q^{n+1}_\text {LA}=q^n_\text {LA}+\theta _1 (q^n_\text {PC}-q^n_\text {LA})+\theta _2 (q^n_\text {PC}-q^{n-1}_\text {PC}) \end{aligned}$$

where $0\le \theta _1,\ \theta _2\le 1$. We then apply $q^{n+1}_{LA}$ as the inflow boundary condition in the LA-MV model to obtain the pressure, $p^{n+1}_\text {LA}$. The pulmonary circulation model takes $p^{n+1}_\text {LA}$ as the pressure boundary condition, i.e.

21 $$\begin{aligned} p^{n+1}_\text {PC}=p^{n+1}_\text {LA} \end{aligned}$$

to advance to the next time step $t^{n+1}$ and obtain $q^{n+1}_\text {PC}$. Different values of $\theta _1$ and $\theta _2$ are used in the numerical implementation to ensure the convergence of the solution. For example, $\theta _1=\theta _2=0.5$ are used during most of the cardiac period except when the MV is closing and the LA pressure experiences rapid changes especially for the AMR case, $\theta _1=\theta _2=0.1$ are used instead. The coupled system then runs for several cardiac cycles until the solutions are convergent to a steady state.

### Physio-/pathological cases

At the inlet of the main pulmonary artery, a physiological RV pressure is applied together with a lumped parameter model representing the normal function of the pulmonary valve (Mynard et al. 2012) which does not allow regurgitation. At the downstream of the coupled system, the LV function is also mimicked using a pressure boundary condition. Another key boundary condition is the movement of the chordae tips which stands for the contraction and relaxation of papillary muscles. In the current work, the movement is achieved using a time dependent displacement boundary condition based on the measured data from Wang and Sun (2013).

**Fig. 5:**
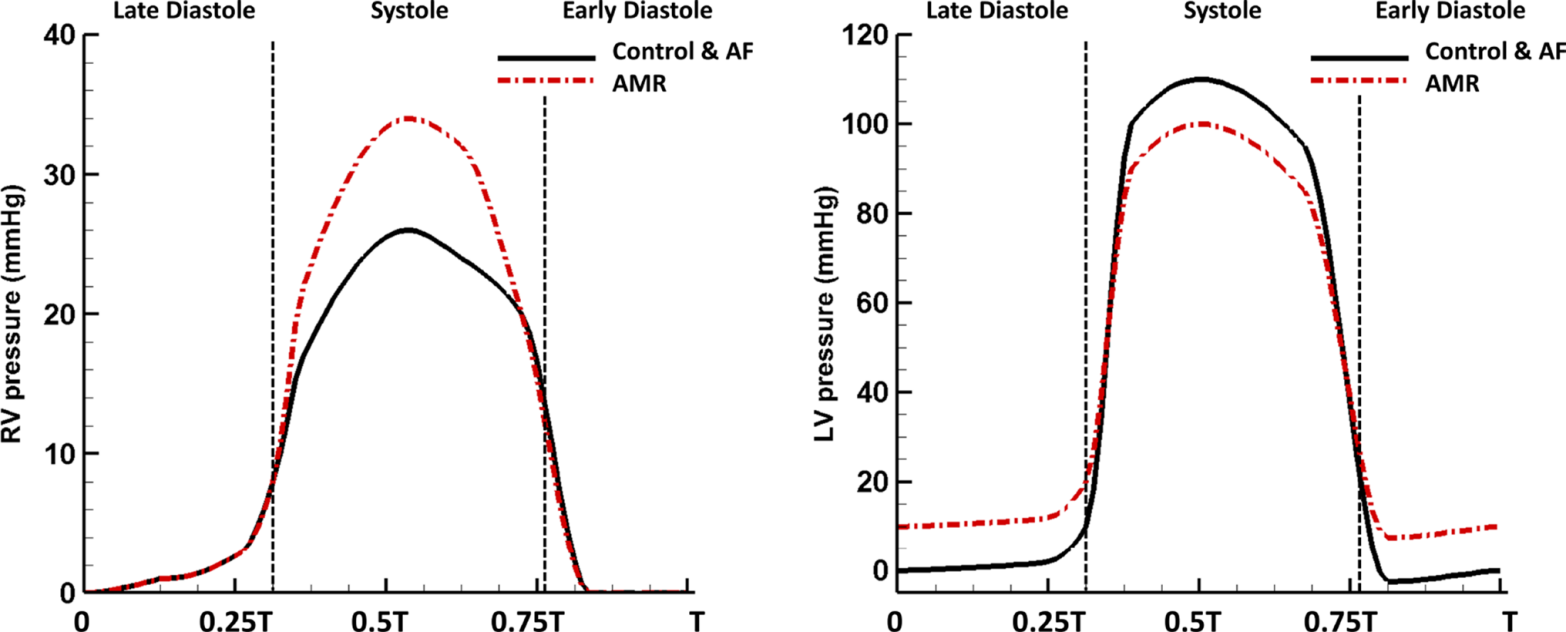
Right and left ventricular pressure boundary conditions in three cases. *T* represents the cardiac period which equals to 0.8 s in the control and AF cases, and 0.6 s in the AMR case. *RV* right ventricle, *LV* left ventricle

Three different cases are studied using the fully coupled model:

1. *The control case*: the normal LV and RV pressure profiles from literature (Klingensmith et al. 2008) are applied, as shown in Fig. 5, with the cardiac cycle set to be 0.8 s (75 bpm). In this study, systole (duration 0.36 s) and diastole (duration 0.44 s) are referred to the ventricular systole and diastole, respectively. In this study, we assume both ventricles contract and relax simultaneously, and the ventricular pressure profiles follow the similar pattern to mimic the synchronized RV and LV dynamics, and the synchronization to the LA-MV apparatus is achieved via imposing the LA active contraction (duration 0.12 s) at the end of diastole and the MV chordae tips movement at the beginning of the systole and the end of the systole to facilitate the MV closing and opening. The normal chordae tips movement are included based on the MSCT image data from Wang and Sun (2013).
2. *The AF case*: the loss of atrial active contraction is mimicked by removing the active stress tensor in (12). The rest of the boundary conditions are the same as in the control case. We understand this is a simplified AF case. In clinical situations, the atrial chamber with AF may have a chronic dilation (Blume et al. 2011), and the atrial pressures throughout the cardiac cycle may also be elevated. These extra complications will not be addressed here.
3. *The AMR case*: the acute mitral regurgitation is achieved by altering the normal chordae tips movement during systole to obtain valve incompetence. Additionally, AMR patients often suffer from the following abnormalities: increased heart rate (tachycardia) as a compensatory mechanism for the decreased cardiac output per heart beat (Stout and Verrier 2009), increased LV end-diastole pressure and decreased systolic pressure due to the regurgitant MV (Gaasch and Meyer 2008), and increased RV systolic pressure to overcome the increased afterload in the pulmonary system (Nagel et al. 1996). We therefore adjust the pressure boundary conditions (Fig. 5) to reflect these clinical observations while to ensure the same cardiac output per minute as in the control case.

The applied pressures for the three cases are listed in Table 5. It is worthwhile to emphasize that the AF case and the AMR case in the current study are both designed to investigate the early stage pulmonary haemodynamic changes caused by the ‘sudden’ alterations to the normal left heart function without considerations of tissue growth and the remodelling process.

**Table 5:** Pressure boundary conditions for the control, AF and AMR cases

	Control/AF	AMR
Cardiac period (s)	0.8	0.6
RV systolic pressure (mmHg)	26.0	34.0
LV end-diastole pressure (mmHg)	10.0	20.0
LV systolic pressure (mmHg)	110.0	100.0

### Numerical implementation

The LA-MV model is immersed in a viscous fluid with density $1.0\times 10^3$ kg m$^{-3}$ and dynamic viscosity $4.0\times 10^{-3}$ kg m$^{-1}\,$s$^{-1}$ which are the same values used in the pulmonary circulation model. Based on consistence and convergence studies, a two-level block-structured adaptively refined grid with the finest grid spacing 0.125 cm $\times$ 0.125 cm $\times$ 0.125 cm is used for the fluid mesh with 1,843,072 elements. The structural mesh size is around 0.1 cm for the element edge length with 199,511 elements and time step size for the control and AF cases is $6.1\times 10^{-6}$ s and for the AMR case is $4.6\times 10^{-6}$ s. The pulmonary circulation model has the grid spacing 0.05 cm and the time step is chosen to be $4.9\times 10^{-5}$ s for the control and AF cases and $3.7\times 10^{-5}$ s for the AMR case. The time step is chosen to facilitate the fast Fourier transform (FFT) algorithm used to obtain the exact solution for Eqs. (6), (7) and (8) for the small vessel flow dynamics with more details in Qureshi et al. (2014), and the mesh density is chosen following a mesh convergence test shown in Sect. Mesh convergence test in ‘Appendix’. The coupled system is run for several cardiac cycles, and the results are converged to a steady stage at the third cycle from which the results are presented in Sect. 3. All simulations are run on the Cray XC30 supercomputer provided by ARCHER UK National Supercomputing Service (http://www.archer.ac.uk). One cardiac period takes about 48 hours in wall-clock time on two 2.7 GHz, 12-core E5-2697 v2 (Ivy Bridge) series processors.

## Numerical results

### Pulmonary circulation

**Fig. 6:**
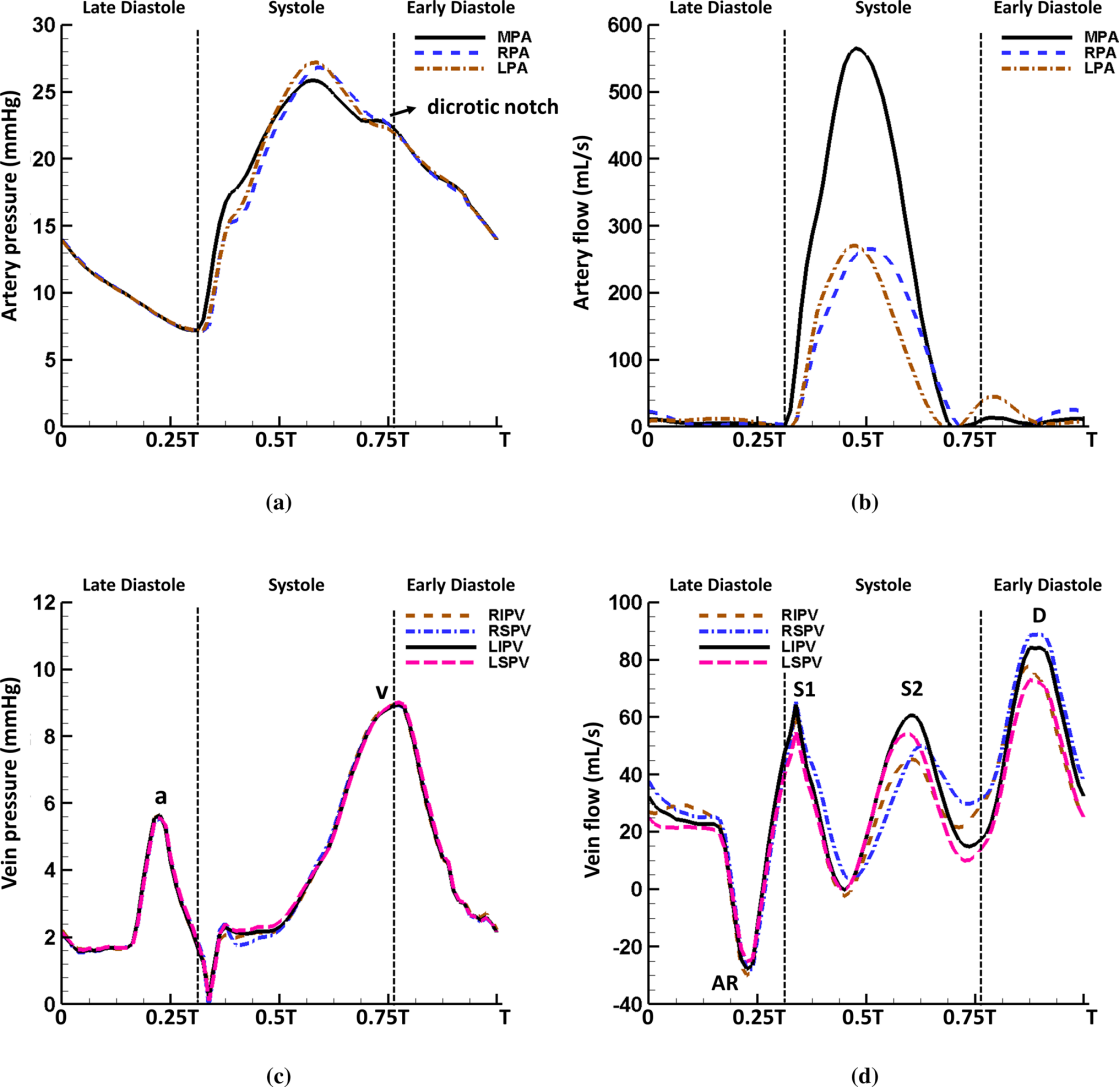
Pulmonary pressure waveforms at the centre of the **a** large arteries and **c** large veins, and the pulmonary flow waveforms at the centre of the **b** large arteries and **d** large veins in the control case with $T=0.8$ s

In Fig. 6a, a typical pulmonary artery pressure waveform is seen in the MPA (for vessel abbreviations, please refer to Fig. 1) with the systolic peak pressure of 26.8 mmHg and the end-diastole pressure of 7.2 mmHg. The dicrotic notch appears at the end systole which indicates the closure of the pulmonary valve as suggested in Fig. 6b. The shift of the pressure waveform in the downstream arteries reflects the wave propagation along the pulmonary vessels. In the large pulmonary veins, the pressure waveform in the control case is similar to a typical LA pressure profile, which includes an end-systole wave (known as the ‘v’ wave) indicating the LA filling and an end-diastole wave (known as the ‘a’ wave) caused by the LA contraction (Fig. 6c). The flow profile (Fig. 6d) in the pulmonary veins captures four different waves: the first systolic wave (S1) caused by the LA relaxation after the active contraction, the second systolic wave (S2) partially due to the upstream wave propagation (Fig. 6b), an early-diastole wave (D) due to the MV opening, and an end-diastole atrial reversal wave (AR) caused by the LA contraction. Since the S2 flow wave reflects the wave propagation through the pulmonary vessel network, differences in the S2 wave arrival times are observed among the four veins and the RSPV shows the latest S2 wave arrival time.

**Fig. 7:**
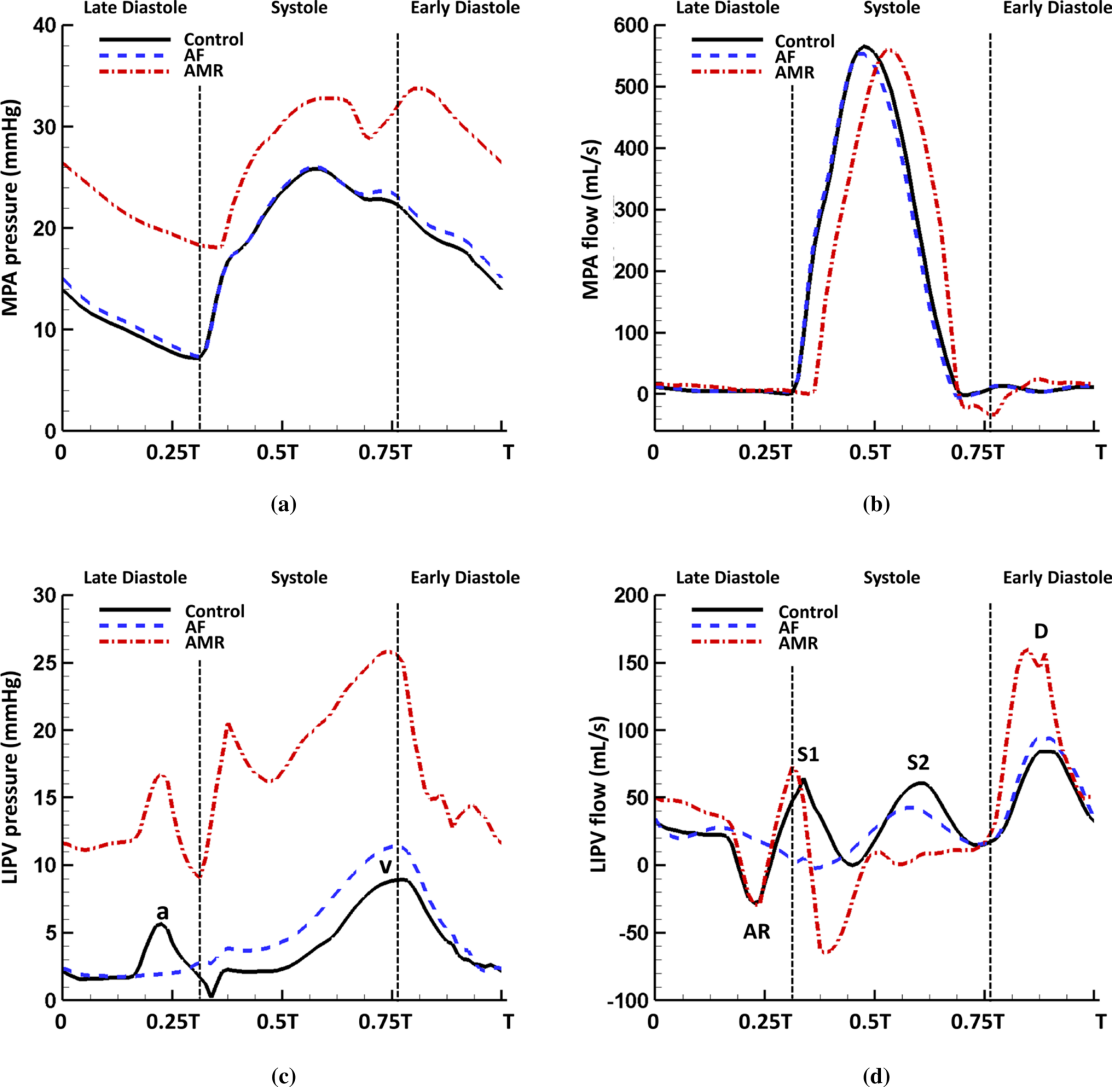
Comparison of the pressure waveforms in **a** the MPA and **c** the LIPV, and flow waveforms in **b** the MPA and **d** the LIPV in the control ($T=0.8$ s), AF ($T=0.8$ s) and AMR ($T=0.6$ s) cases. Notice the mitral regurgitation leads to elevated pressure in pulmonary vessels, reversal venous flow wave during systole and significantly increased D wave. The loss of LA contraction is associated with the disappearance of venous pressure ‘a’ wave, venous flow AR wave and S1 wave

**Fig. 8:**
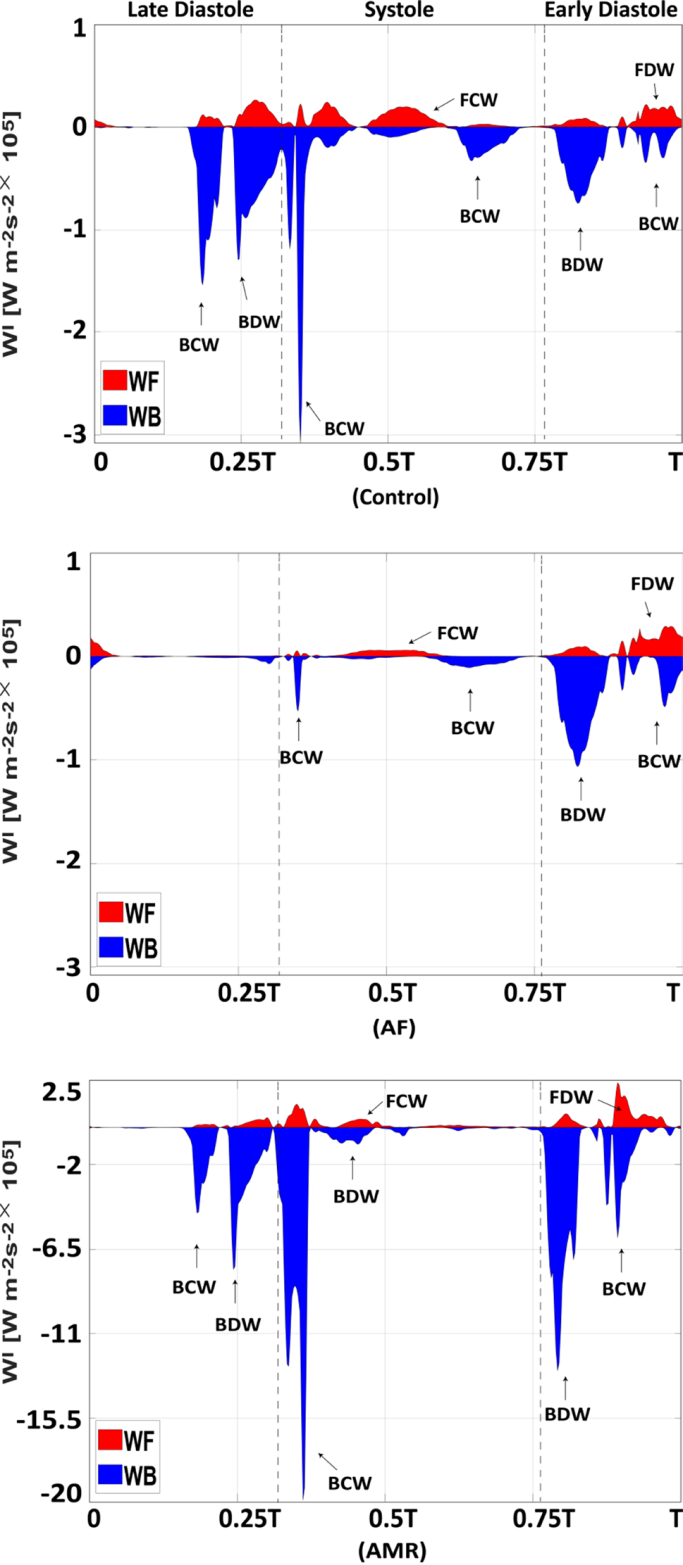
Wave intensity ($\text {W}^{\text {I}_\pm }_i$) profiles at the midpoint of LIPV over a cardiac period in the control ($T=0.8$ s), AF ($T=0.8$ s) and AMR ($T=0.6$ s) cases. WF: wave intensity of forward travelling waves. WB: wave intensity of backward travelling waves. Notice that the LA active contraction generates a backward compression wave (BCW) and the LA relaxation generates a backward decompression wave (BDW). MV closure and opening also produce a backward compression wave (BCW) and backward decompression wave (BDW), respectively. Forward travelling waves have dominant effect at mid-systole and early diastole. In the AMR case, the wave intensities are significantly increased

**Fig. 9:**
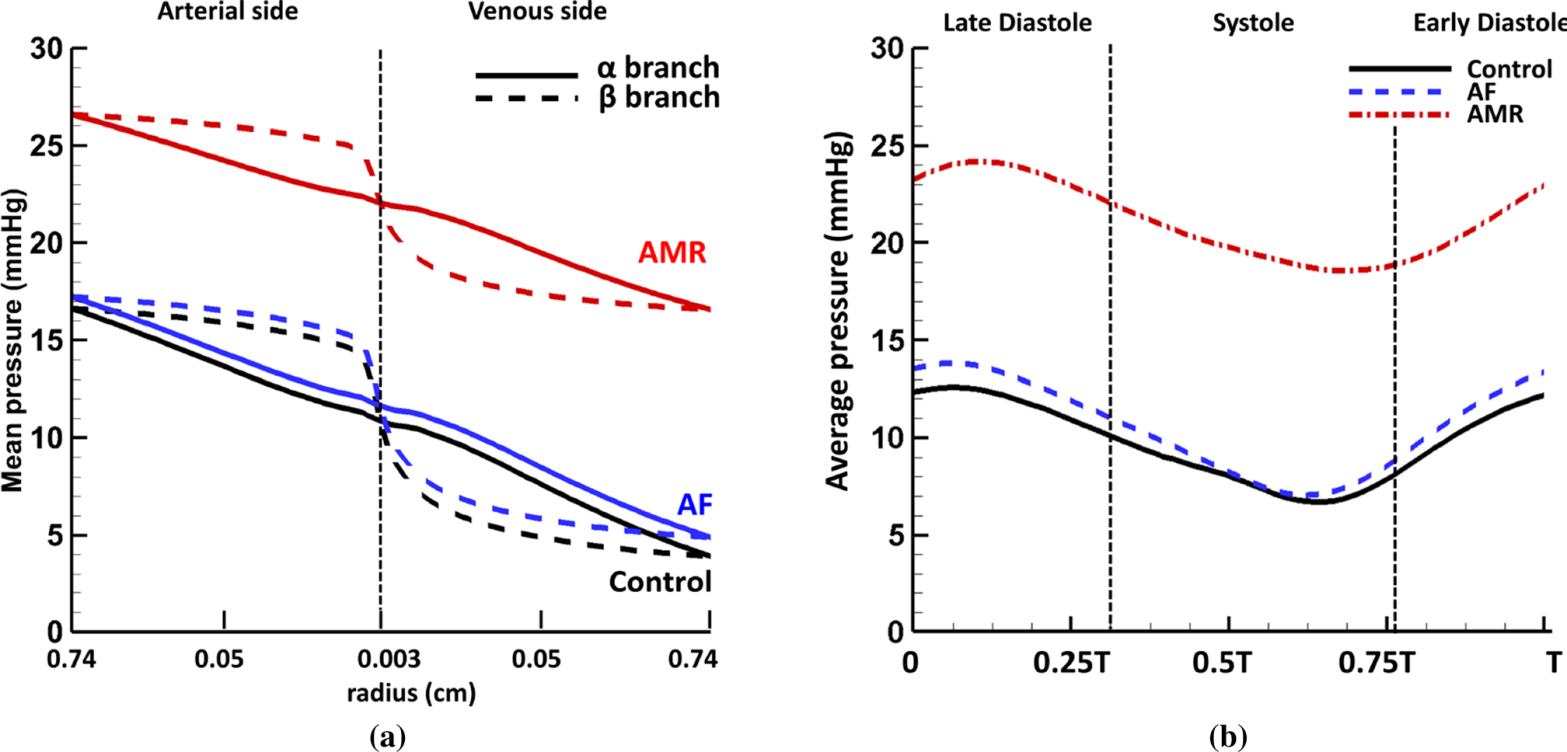
**a** Mean pressure drop along the $\alpha$-only and $\beta$-only branches in the structured tree connecting the LIA and the LIPV. The radius is shown on logarithmic scale (i.e. $\log _{10} r$). Notice the most of the pressure drop happens in the smaller vessels in the $\beta$ branches compared with the $\alpha$ branches. **b** The average pressure in the smallest vessels ($\text {r}=30$ $\upmu$m) in the control ($T=0.8$ s), AF ($T=0.8$ s) and AMR ($T=0.6$ s) cases. Notice the smoother pressure waveform and also the smaller difference between the maximum and the minimum values compared with the typical pressure profiles seen in the large pulmonary vessels (Fig. 6)

Figure 7a, b compares the MPA pressure and flow in the three cases. Overall, the control and AF cases have very similar pressure and flow profiles. However, in the case of AMR, the MPA pressure is significantly elevated with a mean pressure of 26.8 mmHg. The systolic pressure rise in the MPA is caused by an increased RV pressure in order to overcome a greater afterload in the pulmonary system due to a higher LA pressure. The diastolic pressure rise in the MPA reflects an increased LV diastolic pressure as shown in Fig. 5. We also note the large early-diastole pressure wave after the dicrotic notch in the AMR case compared with the control case. Figure 7c, d compares the pressure and flow in the LIPV for the three cases. In the AF case, without the LA active contraction the systolic LIPV pressure is slightly elevated, leading to a reduced S2 flow wave. The AR flow wave and the S1 flow wave also disappear in the AF case. In the case of AMR, the S1 flow wave remains and the LIPV pressure increases rapidly at first at early systole due to the MV regurgitant jets, and then the following pressure decrease is the result of the large backward flow at early systole as shown in Fig. 7d. The MV regurgitation also leads to a suppressed S2 flow wave and a significantly increased D flow wave.

Using a wave intensity analysis, we decompose the waves inside the pulmonary veins into a forward compression/decompression wave (FCW/FDW) and a backward compression/decompression wave (BCW/BDW). The wave intensities ($\text {W}^{\text {I}_\pm }$) are defined as

22 $$\begin{aligned} \text {W}^{\text {I}_\pm }=\frac{\text {d} p^{\pm }}{\text {d} t}\frac{\text {d} u^{\pm }}{\text {d} t}, \end{aligned}$$

where ‘+’ denotes the forward travelling wave from pulmonary vasculature to LA and ‘-’ denotes the backward travelling wave from LA to pulmonary vasculature. Setting $u(x,t)=q(x,t)/A(x,t)$, the forward and backward waves can be obtained by decomposing the original pressure and velocity wavefronts from Eqs. (2) and (3) using the method of characteristics (Qureshi and Hill 2015)

23 $$\begin{aligned} \text {d} p^{\pm }= & {} \frac{1}{2}\left( \text {d} p\pm \rho c\ \text {d}u\right) \end{aligned}$$

24 $$\begin{aligned} \text {d} u^{\pm }= & {} \frac{1}{2}\left( \text {d} u\pm \frac{\text {d} p}{\rho c}\right) \end{aligned}$$

where $\text {d} p$ and $\text {d} u$ represent the pressure and velocity changes over the time interval $\text {d} t$, and *c* denotes the pulse wave velocity obtained via

25 $$\begin{aligned} c=\sqrt{\frac{A}{\rho }\frac{\partial p}{\partial A}}= \sqrt{\frac{2 E h}{3\rho R_0}\sqrt{\frac{A}{A_0}}} \end{aligned}$$

with the help of Eq. (4). Table 6 lists the pulse wave velocity for all large vessels calculated using the mean pressure over one cardiac cycle. The forward and backward waves are further divided into compression ($\text {d} p > 0$) and decompression ($\text {d} p < 0$) waves. Figure 8 plots the wave intensity profile in the LIPV in three cases. At end diastole, the LA active contraction generates a backward compression wave (BCW) followed by backward decompression waves (BDW) due to the LA relaxation. At early systole, a large backward compression wave (BCW) is seen when MV reaches fully closed state and the continuing systolic pressure increase (v wave) is achieved by a dominating forward compression wave (FCW) and a backward compression wave (BCW). At early diastole, the MV opening generates a large backward decompression wave (BDW) followed by a forward decompression wave (FDW), which are responsible for most of the diastolic pressure decrease as shown in Fig. 6c. In the AF case, the late-diastole backward waves disappear and similar wave patterns still remain during systole but with much smaller amplitude compared with the control case. In the case of AMR, the wave intensities are overall significantly increased and an additional backward decompression wave (BDW) appears at early systole corresponding to the early-systole pressure decrease shown in Fig. 7c. Overall, most of the cardiac period is dominated by the backward waves while the forward waves tend to have more contribution at mid-systole and early diastole.

**Table 6:** Pulse wave velocity (m/s) calculated with the mean pressure for the control, AF and AMR cases

Name	Control case	AF case	AMR case
MPA	3.82	3.82	3.93
RPA	3.82	3.82	3.92
LPA	3.82	3.82	3.93
RIA	3.81	3.81	3.92
RTA	3.81	3.81	3.92
LIA	3.82	3.82	3.93
LTA	3.82	3.82	3.93
RIPV	3.69	3.69	3.82
RSPV	3.69	3.69	3.82
LIPV	3.69	3.69	3.81
LSPV	3.69	3.69	3.81

Within the structured vessel trees, the mean pressure (averaged over one cardiac cycle) along small arteries and veins is calculated and the results for the $\alpha$-only (i.e. $r=\alpha ^n \beta ^0 r_{\text {LIA}}$) and the $\beta$-only (i.e. $r=\alpha ^0 \beta ^m r_{\text {LIA}}$) branches connecting LIA and LIPV are plotted in Fig. 9a. The mean pressure drop at the arterial side is about 6.1 mmHg and 6.9 mmHg at the venous side in the control case. Similar to the findings in Qureshi et al. (2014), the vessels in the $\beta$ branches experience higher pressure than those in the $\alpha$ branches at the arterial side and vice versa at the venous side. Additionally, the most of the pressure drop happens in the smaller vessels in the $\beta$ branches compared with the $\alpha$ branches. The AF and control cases have a similar pressure drop, while the AMR case leads to an overall pressure elevation but with reduced pressure drop of 4.8 mmHg at arterial side and 5.4 mmHg at venous side. The pressure waveforms for the smallest vessels with radius 30 $\upmu$m can also be obtained in the current model. For instance, Fig. 9b plots the average pressure over one cardiac cycle in the smallest vessels in the structured tree connecting LIA and LIPV. Compared with the typical pressure waveforms in large vessels shown in Fig. 6a, c, the pressure inside the smallest vessels shows an obvious phase shift and smoother profile. The differences between the maximum and the minimum pressure values are also smaller compared with the large vessels in all three cases, indicating less pressure variation throughout cardiac cycles. .

### Left atrium

Table 7 shows several cardiac parameters over one period in three cases. Stroke volume is defined to be the total blood volume that enters LA chamber from four veins over one period, which can be divided into LA filling volume and conduit volume corresponding to the total blood flow entering LA during systole and diastole, respectively. Overall, all three cases have approximately the same cardiac output 7.5 L/min. Compared with the control case, the large reversal flows in the veins at early systole and disappearance of the S2 flow wave (Fig. 7d) lead to almost zero LA filling volume in the AMR case. Such response appears to prevent the LA chamber from further volume and pressure overloading caused by the mitral regurgitation volume and the elevated LV diastolic pressure. The systolic LA pressure increase is therefore completely driven by the MV regurgitation flow. The loss of active emptying process in the AF case also causes decreased LA filling volume due to slightly elevated LA systolic pressure.

**Table 7:** Several cardiac parameters over one period in the control, AF and AMR cases

	SV	$\text {V}_\text {filling}$ (mL)	$\text {V}_\text {conduit}$ (mL)	$\text {V}_\text {reg}$ (mL)
Control	99.6	45.5	54.1	2.9
AF	96.6	26.8	69.8	2.6
AMR	73.2	− 0.3	73.5	28.7

In the AMR case, notice the LA filling volume is greatly reduced due to the suppressed venous flow shown in Fig. 7d

SV: stroke volume; $\text {V}_\text {filling}$: left atrium filling volume; $\text {V}_\text {conduit}$: left atrium conduit volume; $\text {V}_\text {reg}$: mitral valve regurgitation volume

**Fig. 10:**
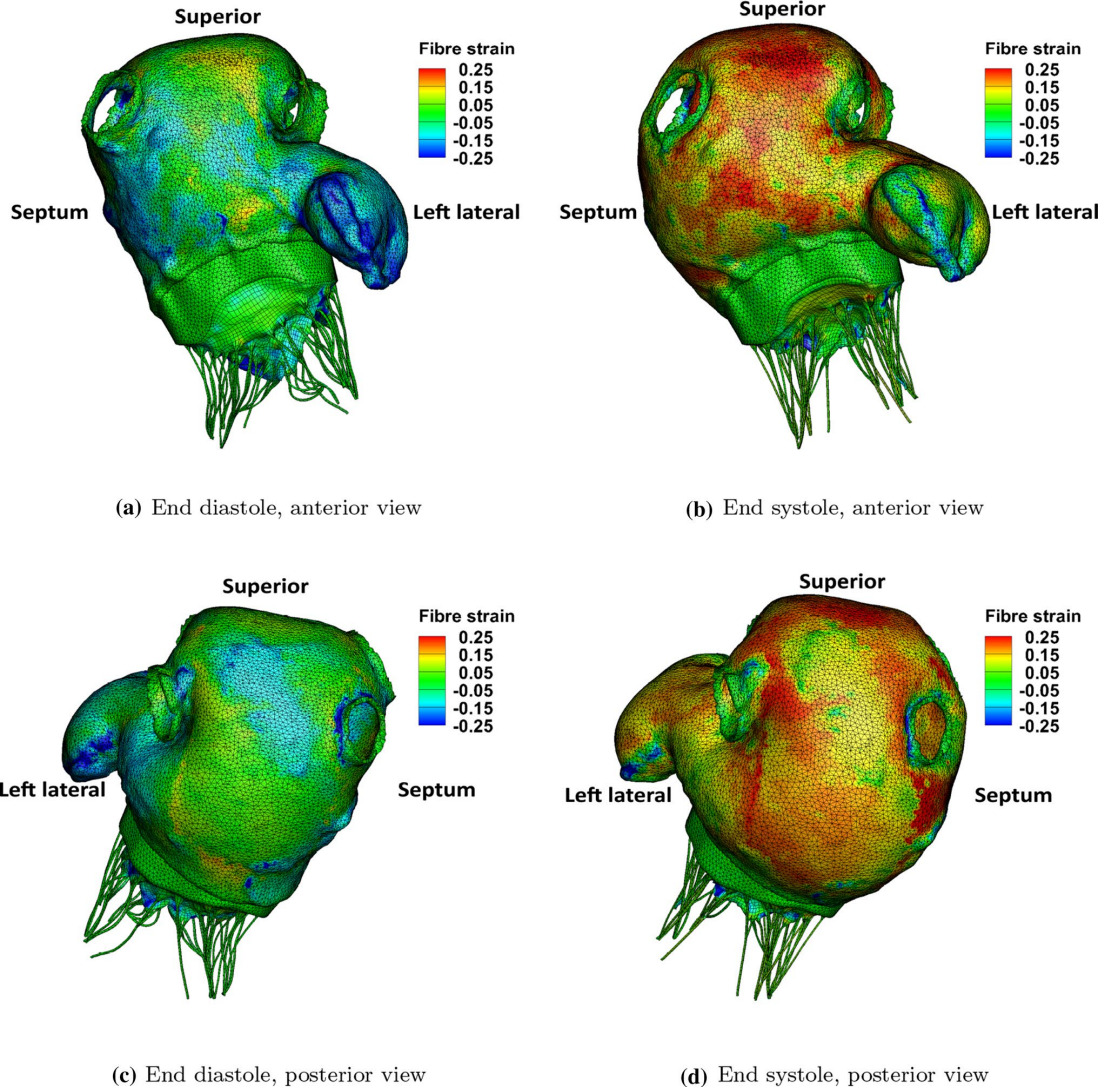
Top row: the fibre strain at the anterior view in the control case. Bottom row: the fibre strain at the posterior view in the control case

**Fig. 11:**
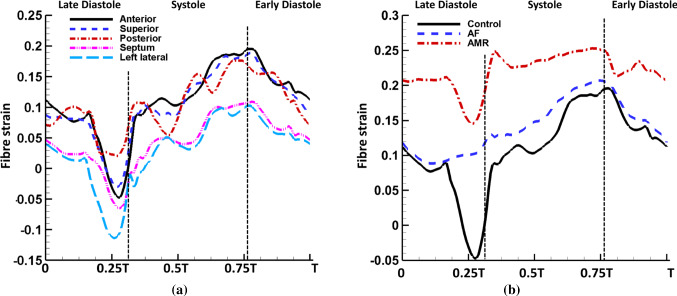
**a** Locally averaged fibre strain on the anterior, superior, posterior, septum and left lateral wall in the control ($T=0.8$ s) case. Notice the anterior, superior and posterior walls have higher strain values than the septum and left lateral walls. **b** The comparison of anterior wall fibre strain in the control ($T=0.8$ s), AF ($T=0.8$ s) and AMR ($T=0.6$ s) cases. Notice the LA active contraction leads to a shortening along the fibre directions as expected (therefore a reduced fibre strain) and the AMR case experiences larger stretch through cardiac cycle

**Fig. 12:**
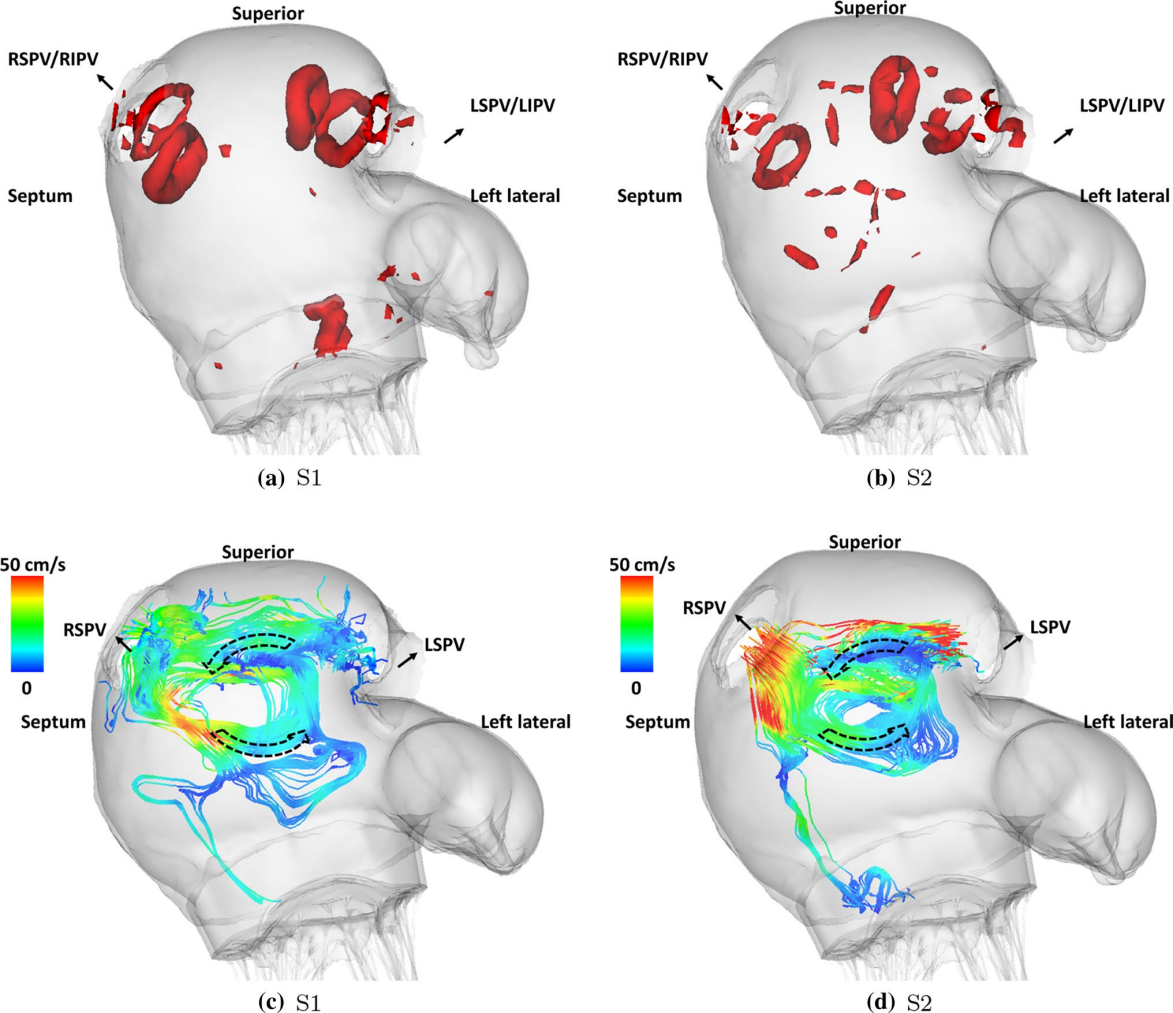
Vortex core detected using the so-called Lambda2 method during **a** S1 flow wave ($\text {t}=0.28$ s) and **b** S2 flow wave ($\text {t}=0.47$ s) in the control case. And the streamlines with origins at LSPV and RSPV orifice at the end of **c** S1 flow wave ($\text {t}=0.38$ s) and **d** S2 flow wave ($\text {t}=0.54$ s) in the control case. As shown in Fig. 7d, the S1 flow wave arrives at four veins simultaneously forming four vortex rings near the venous orifices and the flow jets then meet to form an anticlockwise vortex as indicated by arrows. However, the S2 flow wave arrives at four veins at different times forming only three vortex rings near the venous orifices except the RSPV

**Fig. 13:**
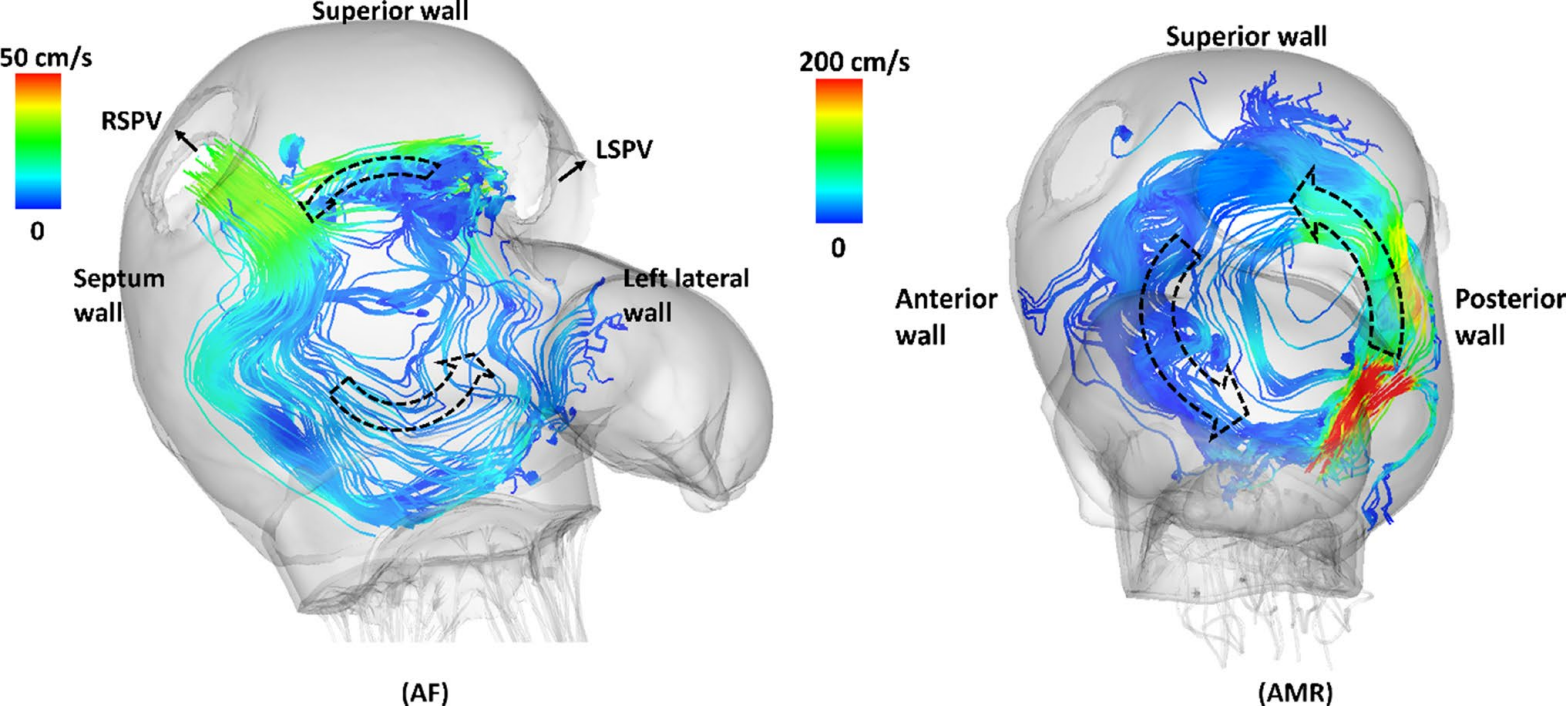
Left: the streamlines with origins at LSPV and RSPV orifice at the end of S2 flow wave ($\text {t}=0.54$ s) in the AF case ($T=0.8$ s). Right: the streamlines with origins at MV leaflets free edges at early systole ($\text {t}=0.41$ s) in the AMR case ($T=0.6$ s)

**Fig. 14:**
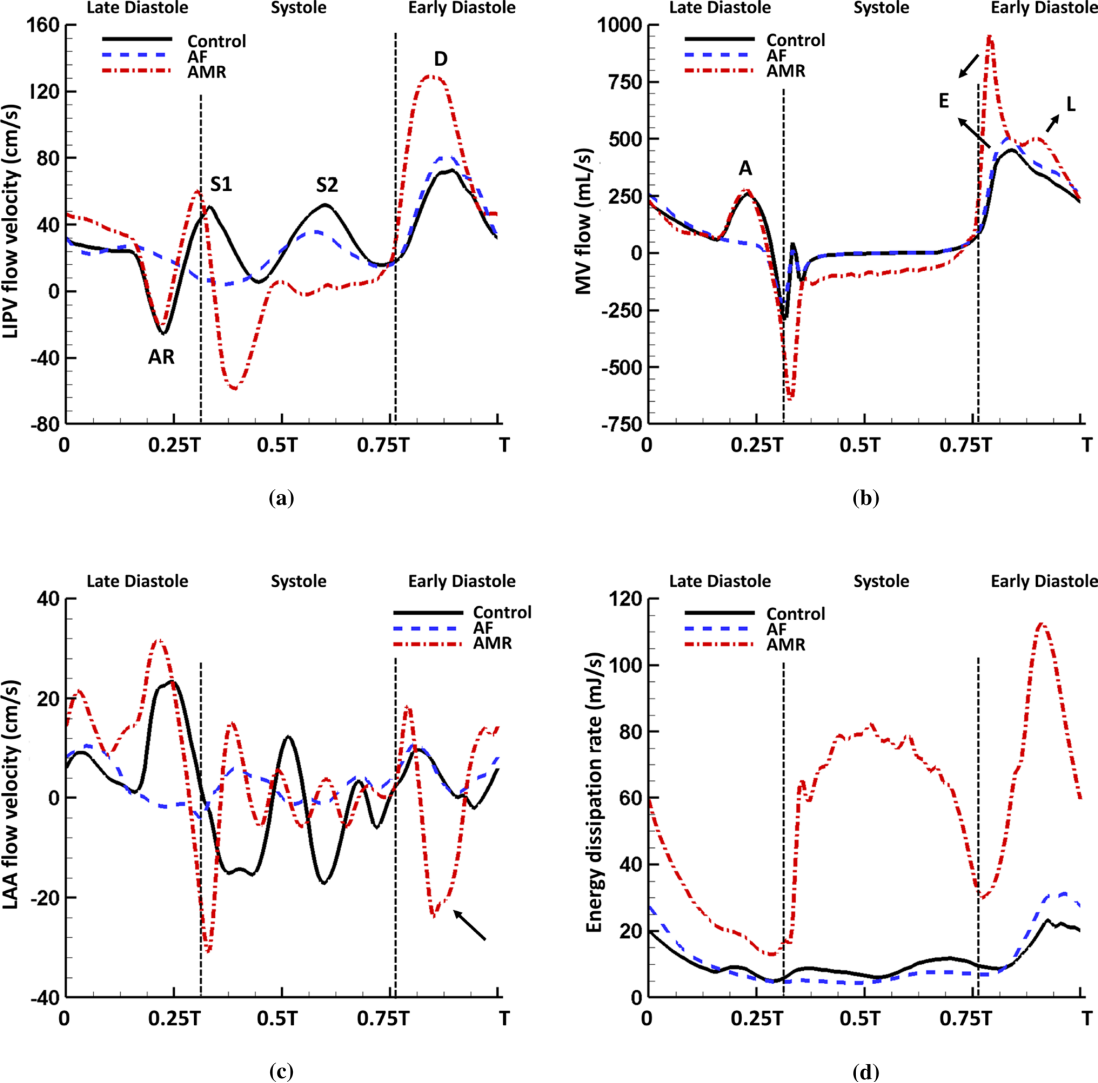
Comparison of **a** flow velocity at the LIPV orifice, **b** flow rate through the MV, **c** the flow velocity at the LA appendage orifice and **d** the energy dissipation rate within the LA in the control ($T=0.8$ s), AF ($T=0.8$ s) and AMR ($T=0.6$ s) cases. Notice the mitral regurgitation leads to increased D wave in venous flow velocity which causes an additional L wave in transvalvular flow and a reversed flow wave in the LA appendage flow velocity during diastole as indicated by the arrow. The AMR case also causes significantly increased energy dissipation compared with the control and AF cases

Figure 10 shows fibre strain on LA walls at end systole in the control case, which is defined as

26 $$\begin{aligned} \text {fibre strain}=\mathbf{e} \cdot \left( \frac{1}{2}\left( \mathbb {F}^{T}\mathbb {F} -\mathbb {I}\right) \mathbf{e}\right) \end{aligned}$$

where $\mathbf{e}$ represents the fibre direction and $\mathbb {F}$ is the deformation gradient. As clinically observed, there is low strain in the atrial wall at end diastole and high strain at end systole. The average fibre strain at the centre of each wall is plotted in Fig. 11a. The anterior, superior and posterior walls have overall higher fibre strain value than the septum and left lateral walls. Figure 11b compares the anterior wall fibre strain in the control, AF and AMR cases. As expected, the late-diastole strain drop, corresponding to the myocyte shortening during active contraction, disappears in the AF case and the fibre strain value in the AMR case is elevated overall. It is interesting to note that the AF case leads to similar end-systole strain as the control case, which can be explained by the reduced LA filling volume balancing the absence of LA active emptying process.

Figure 12 shows the vortices formed inside the LA during systole in the control case. The so-called Lambda2 method is used to detect the vortex ring cores by setting the threshold value to show the most circular ring core (Jiang et al. 2005). During the early systole, vortex ring cores are first formed near the superior wall by the S1 flow jets originating from each vein orifice as shown in Fig. 12a. The flow jets then meet and form an anticlockwise vortex as shown by the two-dimensional streamlines in Fig. 12c. Similar patterns are seen during the S2 flow wave. However, only three vortex ring cores are detected near vein orifices except right superior pulmonary vein (RSPV). This is due to the fact that the S2 flow wave shows different arrival times (Fig. 6d) and the early arrival flow jets from left side veins cause disturbances in the flow field near the RSPV orifice. Compared with the control case, the AF case has similar flow patterns during the S2 wave as shown in Fig. 13a but with a larger anticlockwise vortex. In the AMR case, since the prolapse of the anterior leaflet appears to play a dominant role, the regurgitant jets during the early systole shoot towards the LA posterior wall first and then reach superior and anterior walls leading to a large vortex formation completely different from the control case.

The peak LIPV flow velocity at orifice is plotted in Fig. 14a. Similar to the flow profile, the flow velocity maintains systolic waves (S1 and S2) and diastolic waves (D and AR). Figure 14b shows the flow through MV, which is closely related to the imposed boundary conditions such as the chordae tips movement, LV diastolic pressure and the LA active contraction pattern, etc. The control case has an early-diastole wave (known as the ‘E wave’) with peak 450 mL/s and a late-diastole wave (known as the ‘A’ wave) with peak 261 mL/s. Without the LA active contraction, the late-diastole wave disappears in the AF case. In the AMR case, due to the increased early-diastole pressure gradient between LA and LV, a significantly increased E wave is seen at first. Then, a second early-diastole wave (known as the ‘L’ wave) as indicated by the arrow also appears as a result of the LA refilling caused by the largely increased D wave (Fig. 14a). The increased D wave in the AMR case also has an impact on the flow velocity at the LA appendage orifice, which causes a reversal filling wave after the early-diastole emptying wave as shown in Fig. 14c. Increased wave amplitude is observed for the late-diastole active emptying wave and early-systole filling wave in the AMR case compared with the control case. However, the AF case has much smaller overall velocity indicating blood stasis inside the LA appendage. Figure 14d plots the energy dissipation rate (EDR) caused by fluid viscosity inside the LA chamber

27 $$\begin{aligned} \text {EDR}=\int _{\Omega ^\text {LA chamber}_t}\mu (\nabla \mathbf{u}+\nabla \mathbf{u} ^{T}): \nabla \mathbf{u}\ \ \text {d}\mathbf{x}, \end{aligned}$$

where $\Omega ^\text {LA chamber}_t$ is the flow region inside the LA chamber. In all three cases, a large diastolic wave is captured implying high energy dissipation during LA conduit phase. Additionally, during systole the MV regurgitant jets not only disturb the LA flow field but also significantly increase the energy waste which reflects the low energy efficiency of heart function when MV regurgitation occurs.

## Discussion

In this study, we investigate the interactive behaviour between pulmonary circulation and left heart function under both normal and pathological conditions using computational models. The pulmonary circulation is modelled as a one-dimensional multiscale vessel network, which consists of large arteries and veins as well as small vessels in the form of structured trees. The left heart function is mimicked using a physiologically realistic left atrium and mitral valve model including detailed geometry and material information, and full fluid–structure interaction analysis. The coupled system not only provides insights of the pressure and flow wave propagation through pulmonary vasculature and its impact on the left atrium function, but also reveals the influences of left heart dysfunction on pulmonary haemodynamics in pathological situations of atrial fibrillation and acute mitral regurgitation. To the authors’ knowledge, this is the first study on interactions between pulmonary circulation and left atrium using such detailed computational models.

### The influences of pulmonary circulation on the left heart

Under the normal boundary conditions, the coupled system shows detailed pressure and flow waveforms in the pulmonary vessels (Fig. 6) whose values are in agreement with previous studies. For example, the systolic and diastolic pressure in the MPA in the control case is 25.9 mmHg and 7.2 mmHg, respectively, which agrees with measurements from normal patients by Kane et al. (Kane et al. 2016) (25 ± 5 mmHg for systolic pressure at rest) and Greenfield et al. (Greenfield JR and Griggs JR 1963) (8.1 ± 3.2 mmHg for diastolic pressure). More importantly, the wave propagation through the pulmonary vasculature is found to affect the LA flow field in the coupled model. For example, the forward compression wave (FCW) at mid-systole (Fig. 8a) leads to different arrival times for the S2 flow wave (Fig. 6d) between the veins due to the fact that the forward wave travels through the pulmonary vasculature and different veins are connected to vessel trees with different anatomies. As a result, during the S2 wave, vortex ring cores (Fig. 12) are only formed near the pulmonary veins with early arrival waves. On the other hand, the S1 wave which is ‘left atrium driven’ generates vortex ring cores near all veins due to the same wave arrival time.

In the study by Qureshi et al. (2014) using a single pulmonary circulation model with a constant LA pressure boundary condition, different flow wave arrival times are also seen among large veins for a healthy case. Significantly, in the AF case, we hardly see any different S2 wave arrival times among the veins. This is because the reflections of backward travelling waves (Fig. 8), which are generated by the LA contraction and travel through the pulmonary vasculature, are also important in generating the S2 wave as discussed in Sect. 4.3. Without LA active contraction, this time delay phenomena are not as obvious as in the control case. In the AMR case, there is no S2 wave. To the best of our knowledge, this behaviour of S2 waves presented in our coupled model has not been recognized in literature and could be useful in disease diagnosis.

In the case of AMR, the high LA pressure alters the normal flow patterns inside pulmonary veins which in turn lead to disturbances to LA flow field. For example, the significantly increased early diastolic D flow wave (Fig. 14a) is seen to cause an additional short LA filling period during the LA conduit phase, which results in a reversal flow wave at the LA appendage orifice at the early diastole (Fig. 14c) and a second early-diastole wave at the MV orifice (Fig. 14b), or the mitral L wave (Kerut 2008; Lam et al. 2005).

### The influences of AMR on pulmonary circulation

As mentioned in Sect. 1, the development of PHLHD can be divided into the reversible stage and the irreversible stage. The transition is believed to be triggered by the long-standing high pulmonary venous pressure but not yet fully understood (Patel et al. 2014; Galiè et al. 2016; Guazzi and Borlaug 2012). Our coupled model investigates the reversible stage caused by acute mitral regurgitation and provides more insights into the PHLHD disease progression. At first, the incompetent MV leads to regurgitation jets during systole as shown in Fig. 13, which causes larger systolic pressure increase in the LA chamber compared with the control case. The high LA pressure is then transmitted backwards into pulmonary veins causing a large reversal flow wave during systole and suppressing the S2 flow wave (Fig. 7d) in agreement with the clinical observations (Tabata et al. 2003; Klein et al. 1992). As a result, the LA filling process during systole in the AMR case is dominated by the regurgitant MV flow rather than forward systolic flow coming from pulmonary circulation in the control case as suggested in Table 7. It is worthwhile mentioning that in general the MV regurgitant jet direction and the flow vortex formation within the LA are closely related to the type of mitral regurgitation. For example, a posterior leaflet prolapse will tend to produce a regurgitant jet directed anteriorly along the anterior and septal LA walls, whereas a prolapse of the anterior leaflet will produce a posteriorly directed jet along the posterior LA wall. In this study, the prolapse of the anterior leaflet appears to be the main cause of the observed flow pattern.

In addition to the different wave patterns observed in the large veins, the mean pressure elevation within arterioles, venules and the capillary bed also shows the influences of AMR. In the current study, the mean pressure increases by 11.2 mmHg in the smallest vessels ($\text {r}=30$ $\upmu$m) compared with the control case. However, even though the overall pressure is elevated in the AMR case as shown in Fig. 9a, the total mean pressure drop across the structured vessel trees is reduced to 10.2 mmHg compared to the control case of 13.0 mmHg. This is explained by the increased cross-sectional area of the small vessels due to a higher pressure, while the vessels’ wall stiffness remains unchanged. These findings agree with clinical practice in which the transpulmonary pressure gradient (TPG), i.e. the difference between the mean pulmonary artery pressure (mPAP) and the pulmonary artery wedge pressure (PAWP) which estimates the LA pressure, serves as an important parameter to distinguish the reversible and the irreversible stages of the PHLHD (Galiè et al. 2016). For example, elevated TPG in the case of PHLHD often indicates increased pulmonary vascular resistance and structural changes within the pulmonary vascular bed while in the reversible stage of PHLHD, TPG remains minimally affected. As mentioned in Sect. 1, clinically, pressure change in the small vessels is very important since a rapid pressure increase in the pulmonary capillaries can lead to acute pulmonary oedema (Ware and Matthay 2005), and the high pressure also serves as a triggering factor for vessel structural and functional changes. By looking into the pressure waveform in the smallest vessels (Fig. 9b), our results show that the microscopic vessels experience smoother pressure changes and less pressure variations over cardiac period compared with large vessels. Therefore, the presented model provides a framework to study and predict the haemodynamic changes within the capillary bed where such information can only be indirectly inferred clinically. Finally, AMR induces high pressure in the pulmonary arteries, indicating an increased RV afterload. In the AMR case, another obvious change in the MPA pressure is the appearance of a large wave at early diastole (Fig. 7a) immediately following the dicrotic notch, which is also observed by Grose et al (Grose et al. 1984).

### Wave intensity analysis

Wave intensity analysis (Parker and Jones 1990; Parker 2009) has been widely used to study the wave propagation in blood vessels. However, compared with the pulmonary arteries, much less effort has been put into analysing pulmonary venous waves due to the challenges in obtaining accurate pressure and flow velocity information (Smiseth et al. 1999; Bouwmeester et al. 2014; Hellevik et al. 1999). Smiseth et al. (Smiseth et al. 1999) performed wave intensity analysis using patients’ data collected during coronary artery bypass surgery and showed that the S1 wave acceleration is attributed to a net backward wave due to the LA pressure decrease, while the S2 wave acceleration is caused by the propagation of RV systolic pressure pulse. Our results (Fig. 8) agree with their study and show that the pulmonary venous waves are mostly dominated by backward travelling waves originating from the LA but forward waves from the upstream of the pulmonary circulation have important contribution to generating the S2 wave and increasing the mid-systole venous pressure, as well as decreasing the venous pressure at early diastole. Hellevik et al. (1999) pointed out the importance of backward waves caused by the LA contraction whose reflection plays a key role in generating the systolic forward wave. In our model prediction, removing the LA active contraction leads to the disappearance of the late-diastole backward waves and reduced wave intensity for the forward compression wave during mid-systole (Fig. 8b), which suggests that this systolic forward wave is a combination of the forward wave generated by RV contraction and the reflection of backward waves generated by the LA contraction.

### Comparison with stand-alone models and global lumped parameter models

As mentioned in Sect. 1, the previous computational modelling of pulmonary circulation and left heart is often separated and such models do not capture the interaction between two systems. In stand-alone left heart models, same boundary conditions are often applied at all the pulmonary venous boundaries. However, as our results suggest, the wave propagation through pulmonary vessels can lead to different pressure and flow waves between each pulmonary vein and these venous waves are also closely related to the LA flow field. Furthermore, our model shows that elevated LA systolic pressure obstructs the LA filling by reversing and suppressing the systolic venous flow. As a result, blood ejected by the RV during systole gets ‘stuck’ within the pulmonary vasculature and then rushes out to the LA chamber during early diastole, creating an increased early-diastole D flow wave (Fig. 7d). Such response is under the influences of pulmonary compliance, pulmonary vascular resistance and vessel stiffness, etc. It is difficult to be accurately described in stand-alone left heart models. We remark that, despite the differences, our results do show some common findings between the stand-alone and coupled approaches. For example, similar to our previous observations in the single LA-MV model (Feng et al. 2019), mitral regurgitation leads to larger flow waves at the LA appendage orifice (Fig. 14c) indicating lower thromboembolic risks inside the appendage while the loss of active contraction slows down the blood flow increasing the risk of thrombosis formation. Additionally, both approaches demonstrate that MV regurgitation results in significantly increased energy dissipation during systole (Fig. 14d).

We must remark that lumped parameter models can offer an alternative and much simpler way for studying pulmonary–heart interactions. As mentioned in Sect. 1, the global cardiovascular system using lumped parameter models including both systemic and pulmonary circulation, heart chambers and valves can be constructed much easily compared to the complex three-dimensional heart models. Such models have proven successful in studying wave propagation in the arterial tree (Van de Vosse and Stergiopulos 2011), and arterial–heart interaction (Segers et al. 2003; Frolov et al. 2017). However, lumped parameter models miss important information such as detailed flow pattern, wall deformation like dynamics of LA walls and MV leaflets. On the other hand, advanced models such as the coupled 3D LA-MV model presented here is able to capture the typical LA flow vortices (Fig. 12c, d) during systole which play an important role in maintaining the energy efficiency in flow dynamics from pulmonary veins to LV by producing washing effects to avoid the blood stasis (Suwa et al. 2014; Föll et al. 2013). Furthermore, the coupled model demonstrates the lack of atrial contraction or mitral regurgitation can lead to altered flow pattern at LAA orifice (Fig. 14c) and the increased pulmonary D wave (Fig. 14a) in the AMR case which causes additional reversal LAA flow during early diastole. These findings cannot be captured in the lumped parameter model, but could have important implications for the thrombus formation inside LAA (Kamp et al. 1999; Verhorst et al. 1993). Additionally, the coupled model provides a framework to investigate the tissue growth and remodelling in the LA (Cameli et al. 2017; Messika-Zeitoun et al. 2007) under PHLHD and in individual pulmonary veins under mitral regurgitation (Tabata et al. 2003; Pieper et al. 1996; Klein et al. 1991). More importantly, the significance of developing the presented complex coupled model is that it can be used to bridge the left and right heart function and eventually move towards the more realistic three-dimensional whole heart modelling including both systemic and pulmonary circulation (Quarteroni et al. 2017).

### Comparison with clinical observations

An important step in the computational modelling is the model validation, which verifies the model assumptions and the accuracy of model predictions. Although direct measurements are not available to compare with our model predictions, the simulated results are aligned with clinical observations. For example, the reported peak velocity for the main pulmonary artery systolic flow is 60–100 cm/s (Bouhemad et al. 2008), which agrees with the peak value 60.0 cm/s in the control case. Clinically, transpulmonary pressure gradient (TPG) and pulmonary vascular resistance (PVR) are often used to evaluate the performance of pulmonary vasculature. In the control case, the TPG and PVR have the values of 13.2 mmHg and 1.76 mmHg min/L, respectively, which fall within the clinically reported ranges (TPG: 9 ± 5 mmHg; PVR: 1.5 ± 0.9 mmHg min/L (Handoko et al. 2016)). Tabata et al. (2003) reported that the typical pulmonary venous flow shows tri- or quadriphasic pattern while the severe mitral regurgitation often leads to reversal systolic flow and increased early diastolic flow. These observations also can be found in our model predictions shown in Figs. 6d and 7d. The study by De Marchi et al. (2001) reports the peak pulmonary venous flow velocity at the RSPV for the systolic wave ($57 \pm 18$ cm/s), diastolic wave ($49 \pm 17$ cm/s) and the late diastolic reversal wave ($20 \pm 9$ cm/s). The predicted RSPV flow velocity in the control case gives peak values of 43.8 cm/s in S1 wave, 66.2 cm/s in D wave and 23.4 cm/s in AR wave. The presented intra-atrial anticlockwise vortex patterns in Fig. 12c, d have also been observed in previous studies using clinical tools such as phase-resolved three-dimensional cine phase contrast magnetic resonance imaging (4D-Flow) (Fyrenius et al. 2001; Suwa et al. 2014; Kilner et al. 2000; Föll et al. 2013). In the study by Dahl et al. (2012), they reported MV flow rate measured by magnetic resonance imaging from a 25-year-old healthy male with approximate E wave peak 480 mL/s and A wave peak 200 mL/s. Our coupled model gives similar results for the control case (E wave peak: 450.0 mL/s; A wave peak: 260.1 mL/s), see Fig. 14b. Comprehensive measurements from one patient including the LA, MV and pulmonary circulation are necessary for a thorough validation of this coupled model, which is not available in this study and also very challenging due to invasive nature of certain measurements, such as blood pressure.

### Limitations

We now mention the limitations of this study. In our model, the pulmonary vessels with radius less than 30 $\upmu$m are neglected. Given the small scales and low resistance of the alveolar vessels, this is unlikely to affect the flow in the large vessels in this study, but will be important in future studies of perfusion dynamics. Sheet-flow or other anatomically based models will be incorporated in future work to capture details of the microcirculation around the alveolar-capillary structure. We have also had to make approximations in the model due to the lack of experimental data for patient-specific geometry such as pulmonary vessel measurements, the matching information between the LA and MV and personalised material properties, etc. Furthermore, we have used a simplified active contraction model especially for the AF case, as suggested in previous studies (Dössel et al. 2012; Fastl et al. 2018; Land and Niederer 2018), which could be more complex than modelled here (Zhao et al. 2017; Allessie et al. 2002).

As mentioned in Sect. 2.4, only the acute effects of mitral regurgitation and atrial fibrillation are studied here. Long-term disease progression such as the material property adaptation for the LA wall, the MV and the pulmonary vessel, and geometrical changes including LA dilation and wall thickening are not included, since these are chronic effects as the result of the volume and pressure overloading (Cameli et al. 2017; Messika-Zeitoun et al. 2007). A further extension of the current work is to incorporate tissue growth and remodelling process into our three-dimensional LA-MV model (Niestrawska et al. 2020; Gao et al. 2017b) following the volumetric growth framework (Rodriguez et al. 1994) or the agent-based approach (Zhuan et al. 2019).

Finally, the movement of the mitral annulus during the cardiac cycle (Levack et al. 2012; Ormiston et al. 1981) as well as the prestrain in the MV leaflets (Amini et al. 2012; Rausch et al. 2013) have not been considered here. This is because our focus is to develop a fully coupled model, rather than to conduct extensive physiological applications of the model. Future work shall also incorporate dynamic MV annular movements which is thought to help with MV closure, balanced leaflet stress distribution, and LV filling and relaxation (Schiros et al. 2015; Carlhall et al. 2004; Ormiston et al. 1981).

## Conclusion

We have developed a computational model for the coupling of the pulmonary circulation and left heart, which is the first to include such detailed anatomical and mechanics information. The pulmonary circulation is modelled as a one-dimensional multiscale vessel network including both macroscopic and microscopic vessels, and the left heart is modelled with detailed atrial geometry, mitral valve leaflets and chordae. Both healthy control and pathological conditions of atrial fibrillation and acute mitral regurgitation are considered. Our model captures the wave propagation through pulmonary vasculature and in particular reveals the effect of the second systolic (S2) flow wave on the vortex formation inside the atrial chamber. Significantly, the behaviour of the S2 waves can be very indicative of different pathologies. In the control case, we observe different S2 arrival times in the veins, but the wave completely disappears in the case of mitral regurgitation, whereas the difference in arrival times is reduced in the case of atrial fibrillation. Moreover, acute mitral regurgitation is found to disturb the atrial flow field, increase the atrial wall strain and interrupt the normal atrial filling by reversing and suppressing venous flow during systole. The pressure waveform in the smallest vessels rises in this case, which imposes an increased afterload on the right ventricle. During atrial fibrillation, our results show slower blood flow near the atrial appendage orifice, disappearance of the late-diastole transmitral flow wave and the late-diastole pulmonary venous pressure wave, but otherwise similar pulmonary artery pressure as the control case. The wave intensity analysis in the pulmonary veins also reveals important contribution of atrial contraction on the venous waves. Overall, the coupled model agrees with clinical observations and can provide useful insights into the interaction between pulmonary circulation and left heart function, especially in pathological situations.

## Figures and Tables

**Fig. 15:**
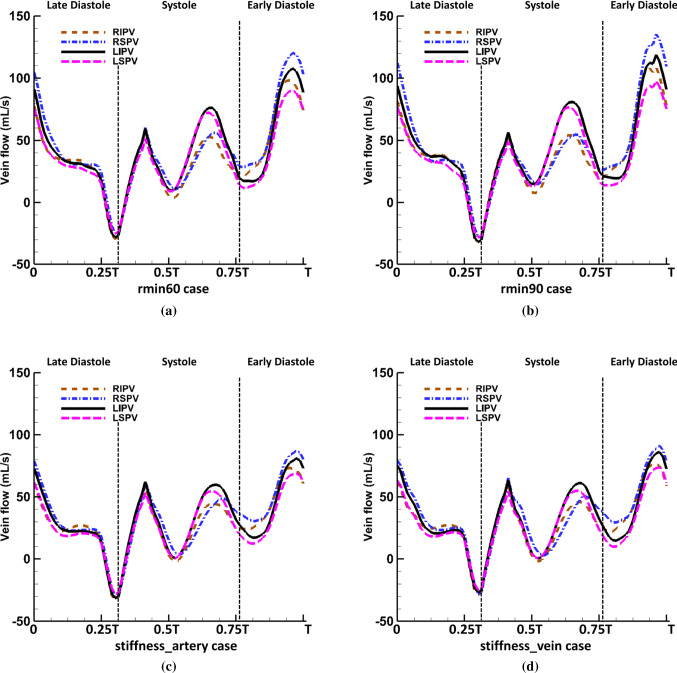
Flow profile in large veins for the rmin60 case, the rmin90 cases, the stiffness_artery case and the stiffness_vein case. The shift of the S2 wave between veins is still present in all cases

**Fig. 16:**
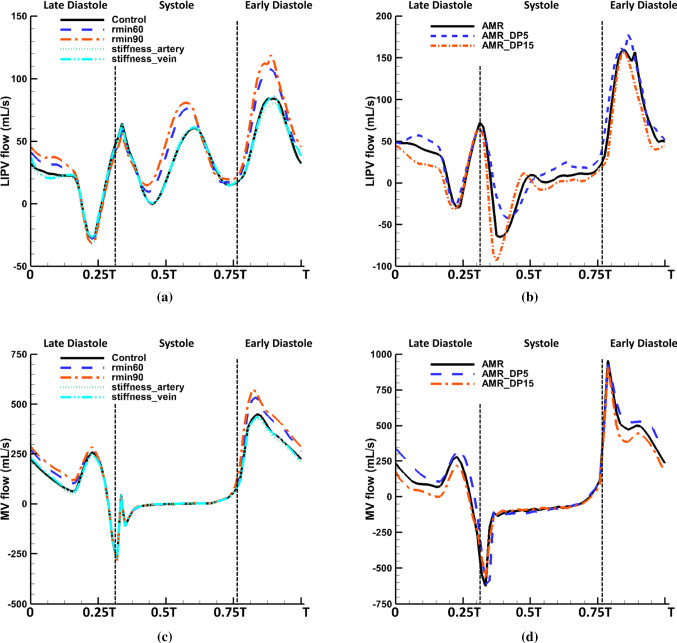
Comparison of **a** LIPV flow and **c** MV flow between the control case, the rmin60 case, the rmin90 cases, the stiffness_artery case and the stiffness_vein case. The comparison of **b** LIPV flow and **d** MV flow between the AMR case, the AMR_DP5 case and the AMR_DP15 case

**Fig. 17:**
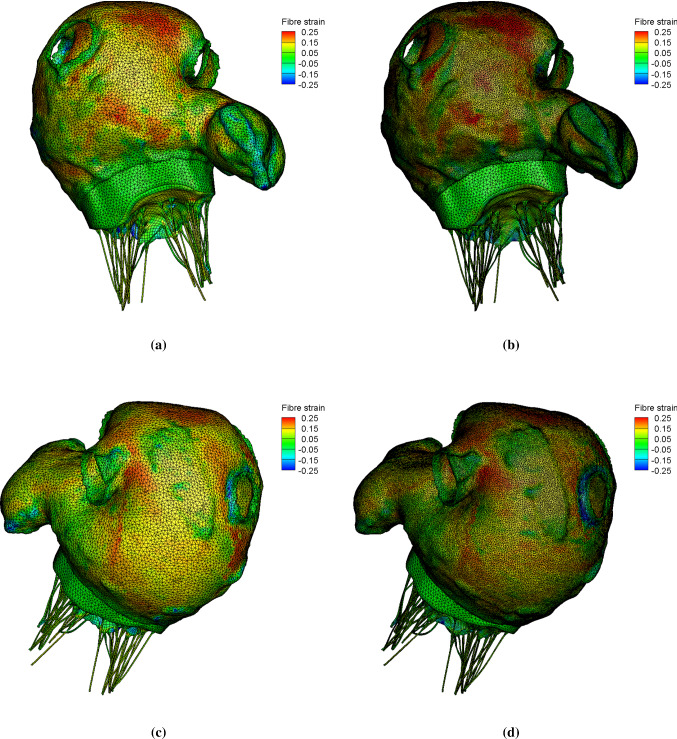
Fibre strain at the end systole for the LA-MV mesh with element edge length of **a** $\Delta x=0.1$ cm and **b** $\Delta x=0.05$ cm at the anterior view, and of **c** $\Delta x=0.1$ cm and **d** $\Delta x=0.05$ cm at the posterior view

## Appendix

### Numerical algorithms

The numerical scheme for the coupled pulmonary circulation model and the LA-MV model is detailed in Algorithm 1.

Let $t^n$ denote the time at the *n*th time step, i.e., $n\Delta t$. In the IB/FE system, the incompressible Navier–Stokes equations are discretized with a Cartesian grid with spacings $\Delta x=\Delta y=\Delta z =h$ and the solid structure is discretized with the first-order linear finite elements. Standard second-order-accurate finite difference approximations are used for divergence, gradient and Laplacian operators denoted by $\nabla _h\cdot$, $\nabla _h$ and $\nabla ^2_h$ (Griffith 2009). ${\mathcal {J}}(\varvec{\chi })$ and ${\mathcal {S}}(\varvec{\chi })$ represent the velocity-restriction and the force-prolongation operators, respectively, defined in detail in (Griffith and Luo 2017). In the IB/FE framework, Eqs. (15) and (16) are only solved on the Cartesian grid. The Eulerian velocity is defined on the element faces and the Eulerian pressure is defined at the element centre. In the current study, the grid size is 120 $\times$ 120 $\times$ 130, and the resultant degrees of freedom for the fluid are 7,533,600. For the solid, a L2 projection problem is solved on the structural mesh with degrees of freedom 200,052.

For the temporal discretization, let $\mathbf{u}^n$, $\varvec{\chi }^{n}$ denote the values for $\mathbf{u}$, $\varvec{\chi }$ at time $t^n$ and $p^{n-\frac{1}{2}}$ denotes the value for *p* at time $t^{n-\frac{1}{2}}$. Basic time-stepping scheme is listed in Algorithm 1. To describe the coupling scheme, let $q^n_{\text {LA}}$ and $p^n_{\text {LA}}$ denote the flow rate and the pressure at the coupling interface (pulmonary vein entries) in the IB/FE system, at time $t^n$. Similarly, $q^n_{\text {PC}}$ and $p^n_{\text {PC}}$ denote the flow rate and the pressure at the coupling interface (pulmonary vein exits) in the pulmonary system, at time $t^n$.

In the pulmonary circulation model, blood flow in the large vessels is solved numerically using Eqs. (2), (3) and (4). In short, the system of equations are first rewritten in the conservation form

28 $$\begin{aligned} \frac{\partial }{\partial t}\mathbf{U}+\frac{\partial }{\partial x}\mathbf{W}=\mathbf{S}, \end{aligned}$$

where

29 $$\begin{aligned} \mathbf{U}= & {} (U_1,U_2)=(A,q), \end{aligned}$$

30 $$\begin{aligned} \mathbf{W}= & {} (W_1,W_2)=\left( q,\frac{q^2}{A}+f\sqrt{A_0 A}\right) , \end{aligned}$$

31 $$\begin{aligned} \mathbf{S}= & {} (S_1,S_2)=\left( 0,-\frac{2\pi \nu R}{\epsilon }\frac{q}{A}+\right. \end{aligned}$$

32 $$\begin{aligned}&\quad \left. \left( 2\sqrt{A}\left( \sqrt{\pi }f+\sqrt{A_0} \frac{df}{dr_0}\right) -\frac{df}{dr_0}\right) \frac{dr_0}{dx}\right) , \end{aligned}$$

in which

33 $$\begin{aligned} f(r_0)=\frac{4Eh}{3R_0}. \end{aligned}$$

*A*(*x*, *t*) and *q*(*x*, *t*) are then discretized with $A^n_m$ and $q^n_m$ representing the values at time $n\Delta t$ and location $m\Delta x$ in each large vessel, and similarly for $\mathbf{U}$, $\mathbf{W}$ and $\mathbf{S}$. Equation (28) is solved using a two-step Lax–Wendroff scheme, with details listed in Algorithm 1.

### Parameter sensitivity test

Parameter sensitivity analysis is an important component in the modelling process to identify the key factors in the selected parameters and boundary conditions which might affect the model performance significantly. In the current model, the model parameters include pulmonary vessel geometry, vessel stiffness, LA-MV geometry and the parameters of LA-MV material models, and the parameters in boundary conditions include systolic-to-diastolic duration ratio, peak systolic pressure values, diastolic pressure values, etc. In this section, we present a preliminary investigation for the effect of different radius cut-off values (i.e. $R_\text {min}$) for the structured tree, vessel stiffness ($Eh/R_0$) and LV diastolic pressure.

Six additional cases are considered here: the rmin60 and rmin90 cases have different cut-off values compared to the control case; the stiffness_artery and stiffness_vein cases have modified vessel stiffness in only large arteries and large veins, respectively, compared to the control case; the AMR_DP5 and AMR_DP15 cases have different end-diastole pressure compared to the AMR case. The details are listed in Table 8.

Here, we show results for the pulmonary venous flow and the flow rate through MV. Figure 15 shows the pulmonary venous flow in four veins for the rmin60 case, the rmin90 case, the stiffness_artery and the stiffness_vein cases. The shift of the S2 wave between veins is still present even though the flow present different peak values compared to the control case as shown in Fig. 16a. The variations in the LV end diastolic pressure for the AMR case lead to similar LIPV flow pattern (e.g. the suppressed S2 and increased D waves) as shown in Fig. 16b.

Figure 16c, d shows the comparison of MV flow rate for all cases. Similar MV flow patterns are still present even with different flow peak values.

**Table 8:** Modified parameters for various cases

Cases	Parameters
rmin60	$r_\text {min}$ = 60 $\upmu$m
rmin90	$r_\text {min}$ = 90 $\upmu$m
stiffness_artery	$Eh/R_0= 225.0$ mmHg for large arteries
stiffness_vein	$Eh/R_0= 75.0$ mmHg for large veins
AMR_DP5	End-diastole pressure is 5 mmHg
AMR_DP15	End-diastole pressure is 15 mmHg

### Mesh convergence test

The convergence and the accuracy of the immersed boundary method with finite element elasticity (IB/FE) have been investigated with details in the previous work (Griffith and Luo 2017; Griffith 2009) and the performance of the numerical schemes used in the pulmonary circulation model (Sect. Numerical algorithms in ‘Appendix’) have also been validated using published studies from the physiological literature (Qureshi 2013).

In this section, we present some results of the mesh convergence test for the coupled model. First, two different grid spacings, $\Delta x=0.05$ cm (used in this study) and $\Delta x=0.025$ cm, are compared in the single pulmonary circulation model with prescribed RV and LA pressure conditions. The two grid spacings lead to almost identical results with a difference of less than $1\%$. Next, different element edge lengths, $\Delta x=0.1$ cm (used in this study with 199,511 elements) and $\Delta x=0.05$ cm (904,272 elements) for the LA-MV geometry, are compared in the coupled model with prescribed RV and LV pressure boundary conditions. One cardiac period is run for both cases, which requires 48 h in wall-clock time using 24 processor cores for the $\Delta x=0.1$ cm case and 144 h in wall-clock time using 120 processor cores for the $\Delta x=0.05$ cm case. Figure 17 shows the comparison of fibre strain at end systole for two cases. Similar strain patterns are found, and the maximum fibre strain values are 0.38 ($\Delta x=0.1$ cm) and 0.39 ($\Delta x=0.05$ cm).
